# Astrocytes as a mechanism for contextually-guided network dynamics and function

**DOI:** 10.1371/journal.pcbi.1012186

**Published:** 2024-05-31

**Authors:** Lulu Gong, Fabio Pasqualetti, Thomas Papouin, ShiNung Ching

**Affiliations:** 1 Department of Electrical and Systems Engineering, Washington University, St. Louis, Missouri, United States of America; 2 Department of Mechanical Engineering, University of California, Riverside, California, United States of America; 3 Department of Neuroscience, Washington University School of Medicine, St. Louis, Missouri, United States of America; Instytut Biologii Doswiadczalnej im M Nenckiego Polskiej Akademii Nauk, POLAND

## Abstract

Astrocytes are a ubiquitous and enigmatic type of non-neuronal cell and are found in the brain of all vertebrates. While traditionally viewed as being supportive of neurons, it is increasingly recognized that astrocytes play a more direct and active role in brain function and neural computation. On account of their sensitivity to a host of physiological covariates and ability to modulate neuronal activity and connectivity on slower time scales, astrocytes may be particularly well poised to modulate the dynamics of neural circuits in functionally salient ways. In the current paper, we seek to capture these features via actionable abstractions within computational models of neuron-astrocyte interaction. Specifically, we engage how nested feedback loops of neuron-astrocyte interaction, acting over separated time-scales, may endow astrocytes with the capability to enable learning in context-dependent settings, where fluctuations in task parameters may occur much more slowly than within-task requirements. We pose a general model of neuron-synapse-astrocyte interaction and use formal analysis to characterize how astrocytic modulation may constitute a form of meta-plasticity, altering the ways in which synapses and neurons adapt as a function of time. We then embed this model in a bandit-based reinforcement learning task environment, and show how the presence of time-scale separated astrocytic modulation enables learning over multiple fluctuating contexts. Indeed, these networks learn far more reliably compared to dynamically homogeneous networks and conventional non-network-based bandit algorithms. Our results fuel the notion that neuron-astrocyte interactions in the brain benefit learning over different time-scales and the conveyance of task-relevant contextual information onto circuit dynamics.

## Author summary

Astrocytes, a non-neuronal cell type, constitute a significant portion of all cells in the brain, yet our understanding of their involvement in neural computation remains limited. In this paper, we use computational modeling to examine how astrocytes may interact with neurons to expand the types of activity and, ultimately, computations that neurons can produce. Two features of astrocytes make them interesting in this context. First, a single astrocyte is able to modulate dozens of neurons and synaptic connections between them. Furthermore, this modulation occurs at slower temporal scales than the neural activity itself, thus meaning that astrocytes could have long-lasting effects on neural activity and connectivity. Our computational models capture both of these features. Using these models, we show that networks with astrocytes can more readily adapt to slowly varying task parameters versus those with neurons alone. We further analyze the models to understand, in mathematical terms, why such effects may arise. Our results shed light on the potential computational role of this common, but enigmatic type of brain cell.

## Introduction

The role of non-neuronal cells such as glia in neural computation has been the topic of increasing interest over the past decade [1–3]. In the mammalian brain, glia comprise a significant proportion of all cells, comparable to that of neurons [4]. However, their functional role has traditionally been viewed as one of maintaining the basic physiological needs of neurons [5–7]. This view has now been repeatedly challenged owing to a decades-long stream of evidence that these cells directly modulate neuronal signaling [8, 9]. The simple premise here is that the computational power of the brain should be conferred by all cells collectively populating the brain, and not merely by neuronal activity [8–11]. That is, the effects of glia on brain function and neuronal activity exist, and hence must matter. This notion opens up richer and more expansive hypotheses regarding the mechanisms underlying brain computation, including ways by which neuromodulation of networks may be achieved and mapped to function.

In the current work, we zero our attention on astrocytes, a prominent type of glial cell within the nervous system. Collective work in the field of astrocyte biology has repeatedly provided evidence on the instrumental role of astrocytes in controlling neuronal functions such as synaptic wiring, synaptic activity, synaptic memory, and neuronal excitability [12–20], reflecting the potential of astrocytes to control key computational loci in the brain. However, directly probing the role of astrocytes in brain computation has been virtually impossible at the experimental level due to limited knowledge surrounding their rules of engagement and signaling mechanisms, combined with their non-binary rules of ‘excitability’, and our inability to specifically target their neuron-bound modulatory functions without affecting their more general ‘homeostatic’ roles. Additionally, the multiplex nature of astrocytes, whereby a single astrocyte is capable of a multitude of inhibitory, excitatory, or modulatory outputs, distinguishes them in general from neurons. Lastly, the incomplete toolkit available to manipulate them exacerbates the challenge. On the contrary, computational neuroscience provides an ideal playground to probe the role of astrocytes in circuit computation by way of mathematical and algorithmic modeling. So far, the absence of a consensus framework on how to conceptualize astrocyte’s contribution to brain computation in a reductionist way has made it difficult to meaningfully abstract astrocyte functions in computational models. Interestingly, a new hypothesis called “contextual guidance” was recently introduced that potentially alleviates these issues [8]. It posits that astrocytes act as a contextual switchboard that actively conveys information about the environment and physiological state of the organism to neuronal networks. More generally, accounting for astrocytes, and other glial, in neural computation theory may close gaps in how neural circuits learn and implement functions in a manner sensitive to context. For example, an extant issue in theoretical neuroscience pertains to how different but functionally overlapping tasks may be embedded in a single neuronal circuit [21–23]. Such a scenario would seemingly require mechanisms by which different neuronal dynamical regimes may be learned and then recruited, in a context/task-dependent fashion. The goal of this paper is thus to introduce computational modeling and analysis to probe how astrocytes may enrich the computational capability of neural circuits toward such objectives.

Astrocytes contain distinct physiological features relative to neurons. They have slow time-scales of activation, on the order of seconds or slower. Indeed, while approximately 9% of spontaneous astrocyte intracellular calcium events have kinetics of hundreds of milliseconds, most calcium events are documented in the time-scale of seconds and astrocytic outputs and responses to stimuli commonly extend over several tens of seconds. This fact makes them easy to dismiss from the perspective of fast computation. However, these slow time-scales of astrocytes may in fact be a computationally-relevant feature in light of the specific ways in which astrocytes interact with neurons. In particular, neural network function is often viewed through the lens of synaptic connectivity, wherein specific synaptic ‘weight’ configurations are associated with different tasks [24–27]. However, a single astrocyte can impinge on dozens of neurons and hundreds of thousands of synapses, and, for decades now, physiological experiments have indicated that astrocytes possess the capability to gate and influence synaptic plasticity [14, 28–30]. This astrocyte-induced synaptic plasticity belongs to a form of meta-plasticity as outlined in [31]. Along these lines, the involvement of astrocytes in meta-plasticity was further theoretically formalized and modeled in [32]. These prior works substantiate the notion that astrocytes can impact important physiological learning processes. In the current paper, we set forth to examine astrocytic meta-plasticity at a network scale that can be linked to complex functional settings.

Such a framework would represent a shift from common conceptualizations of neural computation that rely on homogeneous neural units, and thus explain how information processing mechanisms may be enacted over different temporal scales. This, in turn, may better reconcile models of algorithmic learning with the physiological realities of the brain. In fact, recent work has argued that astrocytes may implement a transformer-like model of attention in multi-task adaptation and learning in feedforward architectures [33]. In [34, 35], it is shown that neuron-astrocyte interactions can lead in turn to distinct patterns of neural activity in working memory tasks through mean-field network model analyses. In [36], neuron-astrocyte interactions are modeled within neuromorphic spiking neural network architectures, also in the context of memory. There, the model is deployed in image recognition tasks via supervised learning, where it is shown that the presence of slow astrocytic calcium signaling can improve memory performance. Other biophysical and phenomenological models of neuro-astrocyte interactions have also been considered [37–39], however, most of these models are focused on one precise astrocyte output or function (such as glutamate release) or on explaining or recapitulating circuit-level phenomena (e.g., neuronal firing rate activity), rather than connecting to higher-level functions. In the current paper, we focus our attention on the *network dynamics* of neuron-astrocyte interactions in a rate-based recurrent network and reinforcement learning scenario. Specifically, we study neuron-astrocyte interactions with a focus on two dimensions: (i) the dynamics of recurrent interplay of neuronal activity and astrocytic modulation, and (ii) the functional salience of such dynamics in reinforcement learning scenarios. The correlation between network dynamics, e.g., vector fields, attractors, etc., and different functions is itself a crucial area of study in theoretical neuroscience [40]. Furthermore, there is recognition that leveraging the multiple time-scales and heterogeneous structures of recurrent neural networks to design models for learning multiple, sequential, and temporal tasks [41–44]. As such, adding astrocytes to traditional recurrent neural network architectures could thus further expand the expressiveness of these networks [45–47]. Our goal here is to further explore this emerging question.

Motivated by the above, we seek to develop and study a simplified dynamical systems model of neuron-astrocyte interaction in order to gain fundamental insight into how the time- and spatial-scale separation between astrocytes and neurons may enrich the repertoire of neural dynamics and activity. Our models are built in a bottom-up fashion, using well-established biophysical paradigms combined with validated theories of astrocytic modulation of neuronal dynamics and synaptic plasticity, such as described above. Furthermore, we seek to understand how astrocyte-driven dynamics may enable learning over disparate time-scales and in context-dependent task scenarios, consistent with the contextual guidance hypothesis [8]. For the latter, we choose to focus on decision-making problems and reinforcement learning (RL) scenarios, given their relevance and ubiquity in algorithmic learning and prior observations that astrocytes can participate in the encoding of reward information [48, 49].

We proceed to formulate a novel bio-inspired dynamical systems model of neuron-astrocyte interactions, and then embed this model in algorithmic optimization frameworks to solve context-dependent bandit tasks. Our major contributions include the dynamical systems analysis of this model, and understanding astrocytic modulation as a pseudo-bifurcation parameter that can switch neural and synaptic dynamics between different dynamical regimes via meta-plasticity. Herein, astrocytes will form a ‘second-order’ modulation on the time-evolution of synaptic weights, resulting in different generative dynamics of neural activity. We furthermore show that the structure and time-scale separation of astrocytes relative to neurons is enabling in terms of learning non-stationary bandit problems, exceeding the learning performance of well-established algorithms in this domain. It is worth emphasizing that our goal is not to introduce specific dynamics to the model and ascribe these to astrocytes. Rather, we ground our model in extant biological evidence regarding neural-astrocyte interaction which can be linked to specific hypotheses from the contextual guidance framework regarding the role of said interactions.

## Results

### Neuron-astrocyte interactions constitute a hypernetwork with multi-scale dynamics

We proceed to develop a reduced model of neuron-astrocyte interaction that captures key aspects of neurobiology while enabling fundamental analysis regarding dynamical expressiveness and links to function.

#### Neuron-astrocyte structure as a hypernetwork

**Fig 1:**
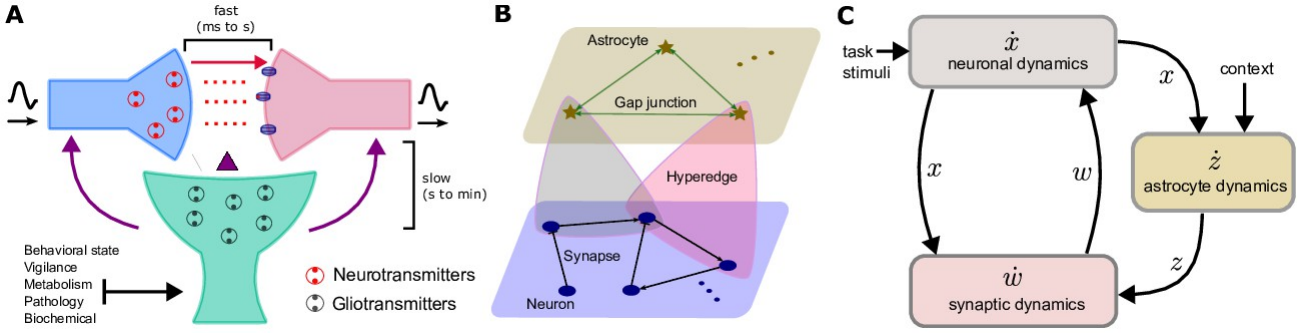
A. In a tripartite synapse, the presynaptic axon and postsynaptic dendrite are surrounded by an astrocyte [11, 50, 51], enabling multifaceted effects of neurotransmitters and (astrocyte-derived) gasotransmitters. B. A graphical illustration of the neuron-astrocyte hypernetwork: the circles and stars represent neurons and astrocytes respectively; the colored triangles denote the hyperedges and represent the multiplexed intralayer interactions. C. Schematic representation of the feedback interconnections between subsystems in the multi-scale neuron-astrocyte network model.

Classically, biological interactions between neurons, astrocytes, and synapses have been conceptualized in terms of the *tripartite synapse* structure [11, 50, 51] (as shown in Fig 1A). Within this framework, astrocytes interact with neurons at synapses, modulating synaptic efficacy [52] and controlling synaptic plasticity [53]. Such interactions may occur in a higher-order and ‘closed-loop’ fashion, wherein astrocytes respond to neurotransmitters released during pre- and post-synaptic neuronal activity (see Section A in S1 Appendix for detailed description) and this has been the mainstream assumption in past work attempting to model astrocytes. While this description may capture an important dimension of neuron-astrocyte interaction, it is increasingly clear that astrocytic modulation of neuronal activity is more general and multifaceted. The *contextual guidance hypothesis* [8] espouses that astrocytes regulate synaptic activity not only in response to synaptic activity itself, but also as an adaptative response to external drives, such as vigilance state, sensory salience, metabolic load, or underlying pathology (see Fig 1A). As such, astrocytes may actively ‘control’ neural dynamics in a state-dependent manner [34, 35]. While we focus here on effects at the synapse via the release of astrocyte-derived neuroactive transmitters, in principle astrocyte modulation can also occur at cell bodies via the alteration of ionic conditions, notably potassium levels [6, 19, 54–56].

The above schema of neuron-astrocyte interactions is difficult to capture as a traditional graphical network representation. As a result, we introduce the framework of a hypernetwork to describe the neuron-astrocyte architecture (see Fig 1B for the illustration and detailed description in Section B in S1 Appendix). We distinguish neurons and astrocytes by representing them on two different layers of the network. The interlayer relationships are all hyperedges, which embody the ability of astrocytes to modulate neuronal synaptic activity and therefore neuronal activity indirectly.

#### Multi-scale neuronal and astrocytic dynamics

The hypernetwork formulation alone does not capture the full complexity of neuron-astrocyte interaction, as it does not explicitly contain information about the time-scales and dynamics of neuronal and astrocyte activation. For this, we introduce a set of ordinary differential equations (ODEs) overlaying the hypernetwork:

$$\tau_{n}{\dot{x}}_{i}=-a_{i}x_{i}+\sum_{j=1}^{n}w_{ij}\phi \left(x_{j}\right)+u_{i},\;i=1,\ldots ,n,$$ (1a)

$$\tau_{w}{\dot{w}}_{ij}=-b_{ij}w_{ij}+c_{ij}\phi \left(x_{i}\right)\phi \left(x_{j}\right)+d_{ij}\psi \left(z_{k}\right),\;i,j=1,\ldots ,n,$$ (1b)

$$\tau_{a}{\dot{z}}_{k}=-e_{k}z_{k}+\sum_{l=1}^{m}f_{kl}\psi \left(z_{l}\right)+g_{k},\;k=1,\ldots ,m,$$ (1c)

$$g_{k}=h_{k}\phi \left(x_{i}\right)\phi \left(x_{j}\right)+v_{k}.$$ (1d)

These dynamical equations are based on firing rate descriptions of neural activity (see Methods for modeling details). Here, *x*_*i*_ describes the rate of the neuron *i* = 1, …, *n*, *w*_*ij*_ is the weight of the synapse (i.e., the synaptic efficacy) between neurons *i* and *j*, and *z*_*k*_ represents the activity (abstracted from calcium activity) of astrocytes *k* = 1, …, *m*. Here we emphasize that *z*_*k*_ embeds a graded but non-linear transformation between the inputs to astrocytes and their output onto neurons. There exist many models for describing the dynamics of neurons, and the one we use is, in essence, a continuous-time rate-based recurrent neural network (RNN) [57]. For the edge weights between neurons, we prescribe a Hebbian plasticity rule wherein weight changes are dependent on the correlation *ϕ*(*x*_*i*_)*ϕ*(*x*_*j*_). The signal *u*_*i*_ conveys external inputs onto neural dynamics.

To distinguish astrocytes from neurons, we use a different activation function (i.e., *ψ*(⋅) ≠ *ϕ*(⋅)) and, most crucially, will assume that the time-scale *τ*_*a*_ is slower than that of neurons. Specifically, a larger value of *τ*_*n*_, *τ*_*w*_, and *τ*_*a*_ implies a slower rate of time-evolution [58] of the associated activity variables. Thus, the multiple time-scale feature of neuron-astrocyte processes is readily captured in equations (1), with a suitable choice of the values of these parameters. Completing the model, *f*_*kl*_ denotes interactions between astrocyte *l* and *k*, allowing for potential gap junctions-mediated communication between neighboring astrocytes [59]. An important feature of the model is that astrocytes may be sensitive to contextual information in accordance with [8], via *g*_*k*_. Here, we postulate two forms of context as specified in (1d). First, we consider a ‘circuit’ context, such that the astrocyte may have a sensitivity of second-order neuronal activity via the coefficient *h*_*k*_. Second, we formulate an external context, motivated by the contextual guidance hypothesis, conveyed by the exogenous ‘contextual signal’ *v*_*k*_. Such a signal may originate, for example, from the sensory periphery [60]. The neuronal exogenous input *u*_*i*_ may also contain such contextual information.

The model above attempts to balance expressiveness, interpretability, and tractability. In particular, we have not fully captured the spatial scale distinctions of astrocytes relative to neurons here, since we restrict ourselves to only the case of two neurons within the domain of a single astrocyte. We have, however, captured several important features of the astrocytic contextual guidance hypotheses: (i) the presence of multiple, nested loops of feedback between neurons and astrocytes, providing a diversity of mechanisms by which contexts can propagate through astrocytes and affect neuronal activity, and (ii) the potentially orders-of-magnitude separation in time-scales between neuronal activity and astrocytic modulation thereof. Unlike previous abstractions such as [34], we do not assume spike-like dynamics within astrocytes, since these cells are electrically inactive on a cell-wide scale. In total, astrocytes modeled here: (a) produce slow, graded activity (as a surrogate for calcium) that (b) modulates neuronal excitability and synaptic plasticity and (c) is responsive to the circuit and external context via feedforward and feedback signaling paths. It is of note that the above neuron-astrocyte model is well-behaved from a dynamical systems perspective since solutions exist, are unique, and are restricted to a bounded subspace (see Section C in S1 Appendix).

From a systems-level perspective, the dynamics of the neuron-astrocyte network can be understood as the interaction between three subsystems, forming two closed-loops as shown in Fig 1C. The first closed-loop consists of the subsystem of neurons (1a) and synapses (1b). The second closed-loop involves the subsystem of astrocytes (1c), which transfers information from neurons to synapses. By forming these closed-loops, the astrocytic process not only directly modulates synaptic plasticity based on neural activity but also indirectly modifies synaptic connections, shaping the dynamics of the network as a whole. This mechanism can facilitate the formation and evolution of attractors (e.g., fixed points) in the neural subsystem state space, as elaborated below.

#### Astrocytic modulation acts as a pseudo-bifurcation parameter that changes meta-plasticity and neural circuit dynamics

To analyze the dynamics of (1), we reduce it to its simplest motif, i.e., the interaction of two neurons and a single astrocyte. Here, we assume that the neurons form a reciprocal excitatory-inhibitory loop, itself a common canonical motif for cortical interactions between pyramidal and inter-neurons. From (1), the neuron-astrocyte motif amounts to a set of 5 ODEs:

$$\begin{array}{c} \tau_{1}{\dot{x}}_{1}=-a_{1}x_{1}+w_{2}\phi \left(x_{2}\right)+u_{1}\left(t\right)\\ \tau_{1}{\dot{x}}_{2}=-a_{2}x_{2}+w_{1}\phi \left(x_{1}\right)+u_{2}\left(t\right)\\ \tau_{2}{\dot{w}}_{1}=-b_{1}w_{1}+c_{1}\phi \left(x_{1}\right)\phi \left(x_{2}\right)+d_{1}\psi \left(z\right)\\ \tau_{2}{\dot{w}}_{2}=-b_{2}w_{2}+c_{2}\phi \left(x_{1}\right)\phi \left(x_{2}\right)+d_{2}\psi \left(z\right)\\ \tau_{3}\dot{z}=-\epsilon z+h\phi \left(x_{1}\right)\phi \left(x_{2}\right)+v\left(t\right). \end{array}$$ (2)

**Fig 2:**
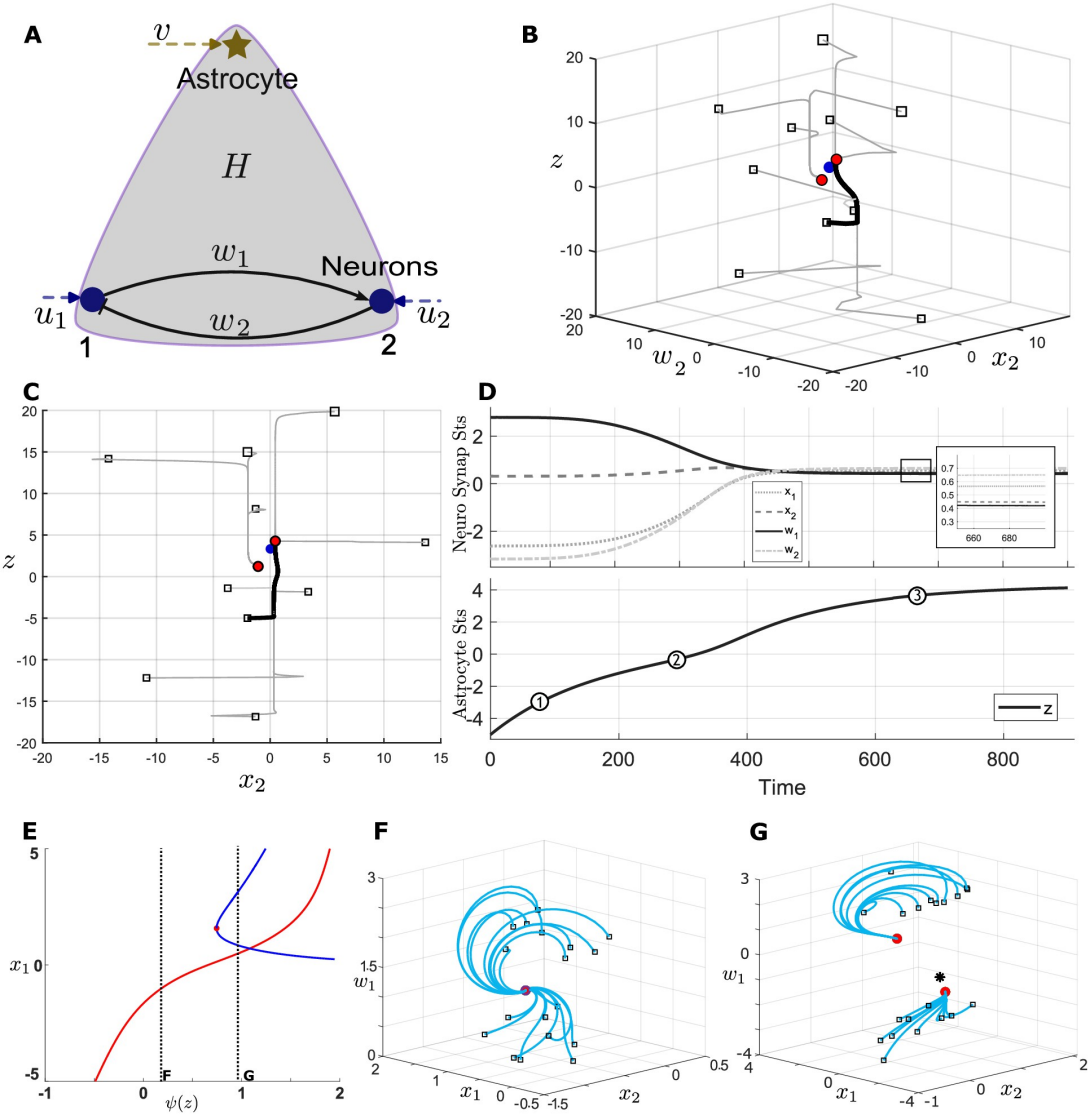
Neuron-astrocyte network motifs and dynamic properties. A. Graphical representation of the network motif; *u*_1_, *u*_2_, *v* include inputs from other nodes of the hypernetwork as well as those from external sources. B, C. Several examples of trajectories in the state space (*x*_2_, *w*_2_, *z*) of the network motif system, where the boxes show the starting points. The parameter conditions are *a*_1_ = 0.7, *a*_2_ = 0.6, *b*_1_ = 1.6, *b*_2_ = 1.7, *c*_1_ = 12, *c*_2_ = −10, *d*_1_ = −4, *d*_2_ = 5, *ϵ* = 0.6, *h* = 6, and *τ*_1_ = *τ*_2_ = 0.01, *τ*_3_ = 1. The system has three fixed point points, of which two are stable (red dots) and one is unstable (blue dot). The system dynamics converge to these two stable fixed points. D. Trajectory associated with the thick phase curve from B, C. illustrating two stationary regimes (indicated by phases 1 and 3 in the lower plot). E. depicts the bifurcation diagram of the neural dynamics with respect to the astrocyte output *ψ*(*z*), where the red curve shows that one branch of fixed point always exists, while the blue curve shows how the other branch of fixed points changes via the saddle-node bifurcation. F, G. Vector fields of the neuron-synaptic dynamics to either side of the saddle-node bifurcation.

The dynamics of this system are asymptotically bounded (see Section D in S1 Appendix). Within this bounded set, the motif may exhibit a unique fixed point, or multiple fixed points, depending on parameterization. Fig 2B and 2C show the case of three fixed points under the assumption that astrocytes evolve at a time-scale two orders of magnitude slower than neurons and synapses (i.e., *τ*_3_ = 100*τ*_1_, *τ*_2_). Fig 2D illustrates the time evolution of a specific trajectory within this landscape. As expected, *z* evolves much slower than the other variables. Notably, this slowly-changing astrocytic activity variable seems to drive neural variables to transit between nearly stationary regimes corresponding to phases 1 and 3 of astrocytic states in the lower plot of Fig 2D, suggesting that astrocytes can systematically ‘control’ stationary neural activity.

In order to understand this phenomenon in more detail, we performed a singular perturbation analysis (see Section E in S1 Appendix) to better clarify the mechanisms by which astrocyte signals may be modulating neural dynamics. This analysis treats the astrocyte state as a fixed parameter, premised on its relatively slow evolution relative to the neural dynamics. We can then study how this parameter affects the vector field and attractor landscape of the neural subsystem. Fig 2E provides the pseudo-bifurcation diagram of the above motif by showing the position of the fixed points in the *x*_1_-dimension as a function of the *ψ*(*z*). When *ψ*(*z*) is small, there is only one fixed point (the red line, also see Fig 2F). When *ψ*(*z*) is large, the neural subsystem manifests three fixed points by means of a saddle-node bifurcation (see Fig 2G). In other words, at the bifurcation point, there is a fundamental change in the shape of the neuronal-synaptic vector field and hence dynamics. Thus, astrocytic modulation can drastically alter the flow of neuronal and synaptic activity as a function of time. We hypothesize this mechanism may be particularly powerful for the contextual guidance premise as it may enable astrocytes to reshape the dynamics of synaptic adaptation and hence neural computation, based on exogenous contextual signals, e.g., via *v*(*t*). Thus, astrocytes form, in essence, a pathway for context-guided meta-plasticity and targeted neuromodulation. Below, we probe this hypothesis within the reinforcement learning task paradigm.

### Neuron-astrocyte networks are able to learn context-dependent decision-making problems

We apply the proposed multi-scale neuron-astrocyte network model to context-dependent decision-making problems. We focus specifically on multi-armed bandits (MABs), a well-known class of reinforcement learning problems, wherein an agent aims to maximize its cumulative reward over time by selecting actions (arms) from a set of available options [61]. MABs find applications in various domains, including recommendation systems, clinical trials, and cognitive tasks in neuroscience, as they provide a powerful framework for decision-making under uncertainty [62]. While well-studied, this class of problems nonetheless poses persistent challenges when environments are non-stationary. Our prevailing hypothesis is that the disparate time-scales of signaling emanating from astrocytes can enable learning in such settings.

**Fig 3:**
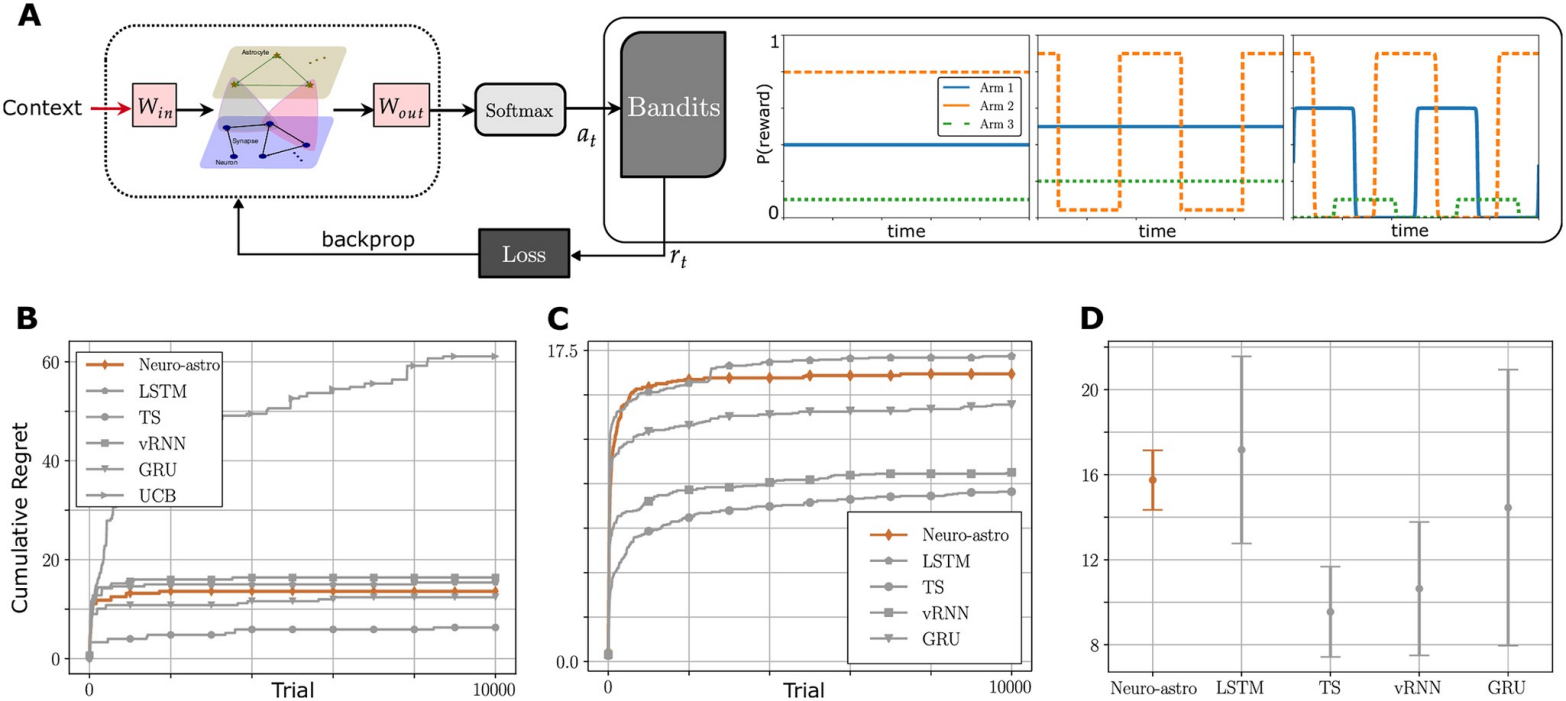
A. The architecture of the learning algorithm. The three plots on the right represent a stationary Bernoulli bandit scenario where the arm means remain (0.4, 0.8, 0.1) constantly over time, a flip-flop non-stationary Bernoulli bandit where Arm 2’s mean alternates between 0.92 and 0.042, and a smooth-change non-stationary Bernoulli bandit where all arm means change according to a smooth periodic function, respectively. The left figure shows the architecture of the learning algorithm. B, C, and D show the learning performance of the neuron-astrocyte (abbreviated as “Neuro-astro” in plots) method relative to other learning methods for the stationary bandit task, where B is for a single run, C is the average result for 10 runs and D is the mean and standard deviation of the asymptotic regrets (the UCB method is not compared in C and D, as it performs much worse than other methods).

A standard MAB assumes a constant environment, in which the probabilities of reward associated with different arms are stationary. Our goal, however, is to study the capacity of our proposed neuron-astrocyte networks, by virtue of their time-scale separation, to learn in more challenging non-stationary and/or context-dependent settings. Thus, we designed both stationary and non-stationary Bernoulli bandit environments (see Fig 3 and Multi-armed bandit tasks in Methods) within which to evaluate learning efficacy.

#### Learning metrics

In MABs, a common figure of merit is the (pseudo) cumulative regret, which is defined specifically in Bernoulli bandits by

$$\begin{array}{c} R_{T}=\sum_{t=1}^{T}\left(\underset{a_{i}\in A}{\text{max}}\;\mu_{i}-E\left[r_{t}\right]\right), \end{array}$$ (3)

where *T* is the total rounds, *μ*_*i*_ is the mean of the action *a*_*i*_, which belongs to the action set A, and *r*_*t*_ is the reward derived by the agent at trial *t* with E[·] denoting the expected value. A lower value of (3) indicates less accumulated loss and equivalently higher accumulated reward. Additionally, we consider the convergence speed of the algorithm, which measures the time taken by the agent for *R*_*T*_ to reach an optimal value. Faster convergence is generally desirable as it signifies more efficient learning by the algorithm.

#### Learning algorithm architecture

In order to evaluate the proposed neuron-astrocyte model in these tasks, we require a learning/optimization method. For this purpose, we make several implementation assumptions. First, we assume that the network emits an output via a softmax operation, a typical form of network readout in neural network architectures. Second, we assume that networks have access to a signal that contains information about the environmental context (e.g., a change in arm probabilities, without overtly specifying the probabilities themselves). Upon this architecture, we deploy a reinforcement learning method to optimize all parameters of the model (see Methods). The architecture of our learning algorithm is depicted in Fig 3A. Briefly, during a typical learning episode, the network outputs a policy for action selection, i.e., a probability distribution over the possible actions (at the output of the softmax). The bandit environment provides a reward to the agent in response, which is then fed into an analytical loss function, for which a gradient can be defined and hence network parameters updated. Crucially, this learning paradigm is agnostic to the specific network being learned, i.e., we can train vanilla RNNs and other architectures with the same methodology. This will allow us to make direct comparisons between the proposed neuron-astrocyte network and other standard neural networks.

#### Performance comparison

We conducted a comprehensive learning performance analysis of the proposed neuron-astrocyte network in comparison to other neural network architectures (vanilla RNN, LSTM, GRU), all trained the same way using the above method. In addition, we also deployed traditional algorithms for solving bandit problems, the Upper Confidence Bound (UCB) and Thompson Sampling (TS) methods. The specific learning procedures for all neural network-based methods are similar, as described in the above section.

**Stationary case**. Fig 3B–3D illustrates the comparison of the learning performance of different methods (neuron-astrocyte, LSTM, TS, vRNN, GRU, UCB) in a stationary bandit task with arm probability settings of (0.4, 0.8, 0.1). Each method requires exploration of the environment, resulting in high regret during the initial time steps. However, all methods eventually converge with comparable rates and cumulative regret of the same order of magnitude. In particular, the neuron-astrocyte architecture performs similarly to the other network-based implementations in this case. Single-run simulation results show that the neuron-astrocyte method uses less time to converge (see Section F.1 in S1 Appendix). In addition, this method tends to be robust as the tasks become more challenging due to the small distance between arm probabilities (see Section F.2 in S1 Appendix).

**Fig 4:**
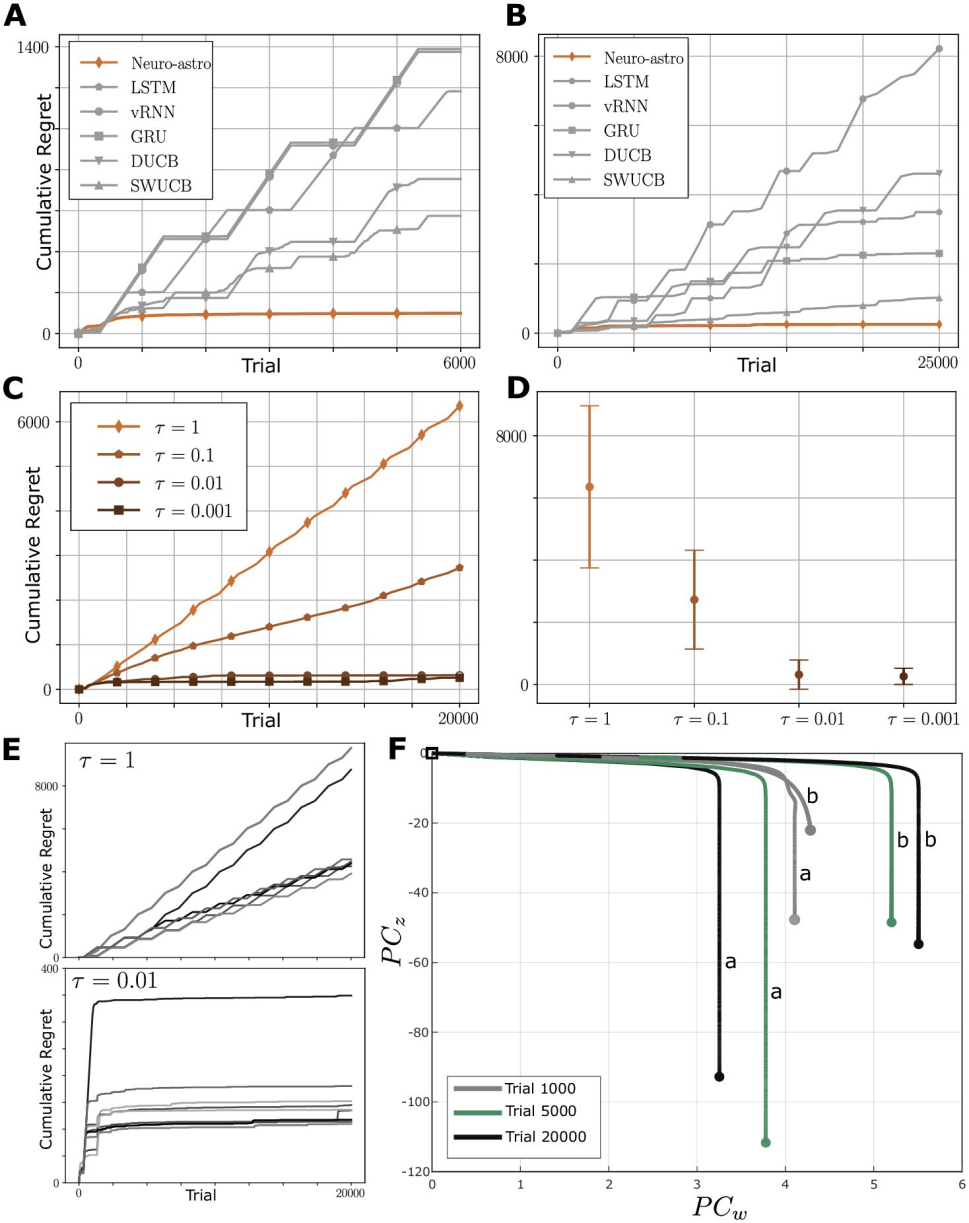
Learning performance. Performance comparison of the neuron-astrocyte method relative to other learning methods for non-stationary bandits: A. flip-flop switching and B. smooth changing. C, D. neuron-astrocyte learning performance for different time-scale separation. E. Single learning traces for *τ* = 1 and *τ* = 0.01, highlighting the role of time-scale separation in enabling RL over contexts. F. Astrocyte and synaptic activity projections for both contexts (indicated as *a* and *b*) in early, middle, and late phases of learning, highlighting the formation of distinct synaptic weight trajectories.

**Non-stationary case**. However, in the presence of non-stationarity, the neuron-astrocyte architecture displays significant gains in capability. Indeed, these networks can achieve almost stationary regrets over time as shown in Fig 4A and 4B, with the former depicting results for the flip-flop bandit and the latter for the smooth changing bandit. In contrast, other methods consistently result in escalating regrets. It is important to emphasize again that the setup for learning here is identical across all networks. These results are consistent across different non-stationary scenarios, evident in both individual and multiple runs (see Section F.3 in S1 Appendix). In addition, similar learning performance is observed in scenarios with a different number of actions (see Section F.5 in S1 Appendix), which suggests the generality of the neuron-astrocyte method. These observations indicate that the neuron-astrocyte network is able to leverage contextual signals and adapt its actions to the changing environment.

#### Time-scale separation is necessary for context-dependent learning

In order to probe the mechanisms by which the neuron-astrocyte network achieves context-dependent learning, we first focus on the time-scale separation between neurons and astrocytes. In our analysis above, we showed how astrocytic modulation may function, in essence, as a form of meta-plasticity wherein the time-scale separation enabled pseudo-bifurcations that could allow neuronal dynamics to traverse different functional regimes. The question at hand is whether this mechanism confers utility for context-dependent learning. To assess this, we varied the time-scale separation (via *τ*) between astrocytes and neurons in our network, to probe the impact of this feature on learning performance. As shown in Fig 4C (see also Section F.4 in S1 Appendix), different *τ* have significant impacts on learning performance, to the extent that without time-scale separation learning simply does not occur. This is seen for the case *τ* = 1, in which astrocytes and neurons have the same time-scale, indicating that the performance of the neuron-astrocyte network does not simply stem from the presence of additional units. Here, the cumulative regret does not converge. When *τ* = 0.1, the agent can sometimes achieve stationary asymptotic cumulative regret. This learning performance improves for greater time-scale separation. For *τ* ≤ 0.01 (that is, time scale separation greater than 2 orders of magnitude), the agent can always adapt to the environment. Moreover, for greater time-scale separation with smaller values of *τ*, there is less variability in the asymptotic regret (see Fig 4D).

To understand the mechanism underlying this effect, we more closely examined the learning dynamics of individual model instances over the different *τ* values, especially the *τ* = 1 and *τ* = 0.01 cases. As shown in Fig 4E, in the case of *τ* = 1, the network is able to learn solutions in each context; however, upon switching, regret again accumulates, indicating an overwriting of prior strategies as comparable to the phenomenon of catastrophic forgetting. On the other hand, neuron-astrocyte networks with time-scale separation are able to reliably learn the flip-flop bandit, indicating that they are able to gradually associate the contextual information with the environment and protect previously learned trajectories. As shown in Fig 4F, the astrocyte-mediated meta-plasticity appears to be engaged during the process of learning. Specifically, we projected the trial-wise network activity along population vectors associated with astrocytes (*PC*_*z*_) and synaptic weights (*PC*_*w*_). We observed that during learning, the network forms distinct synaptic trajectories that asymptotically approach a fixed weight configuration. The time-scale separation between astrocytic and synaptic activation is apparent when tracing the initial stages of the trajectories. The astrocyte output is less sensitive overall to learning, likely an important factor in preventing the context-wise overwriting of prior dynamics (see also Discussion). Furthermore, another projection along the second principal component of astrocytic activity shows that the sign of astrocytic modulation changes across trials and contexts (see Section F.8 in S1 Appendix), which indicates that astrocytic modulations of meta-plasticity can be heterogeneous depending on task circumstances.

## Discussion

### Toward a fuller accounting of brain circuit dynamics

In this paper, we have examined the potential role of neuron-astrocyte interactions in context-dependent learning, with a specific focus on reinforcement-based bandit problems. We began by forming a simplified model of such interactions in the form of a dynamical system, leveraging canonical descriptions of neural firing rate activity and several abstractions of astrocytic activity and modulation that are based on extant neurobiological theory. In particular, we simplified the dynamical description of astrocytes and focused on key aspects: (i) their orders-of-magnitude time-scale separation from neurons, (ii) their modulation of synaptic processes, (iii) their indirect modulation of neuronal firing rates, and (iv) their ability to engage in response to contextual information. Our goal was to understand whether these aspects of neuron-astrocyte interaction, which are known to exist in the brain, matter for network computation.

### Contextually-guided meta-plasticity and slow modulatory dynamics

From this perspective, our analysis indicates the potential for astrocytes to reshape neural and synaptic vector fields in significant ways, such as in the formation of multiple stationary regimes of activation and changing the geometry of synaptic weight evolution. Perhaps most notably, astrocytes can modify the dynamics of synaptic plasticity, effectively switching the network between slow and fast weight adaption regimes (see Fig 4F). This forms a powerful mechanism by which astrocytes can use external and internal contextual information [8] to shift networks between different modes of learning, which we view as a form of meta-plasticity in the wide sense of that term.

One new and central premise to our work is the use of a contextual signal that is accessible by astrocytes and neurons, with the premise that such a signal may embed task-relevant information and/or other circuit contexts, which is highly consistent with the body or work showing astrocytes ability to detect and respond to functionally salient physiological covariates such as neuromodulators (e.g., dopamine), hormones (e.g., glucocorticoids), or local cytokines. Our abstraction of this signal may be viewed as overly strong, insofar as it presents ‘clean’ context information to the network. From this perspective, we emphasize that all our alternative architectures, and especially the neuron-astrocyte model without time-scale separation, had access to this information. Thus, it is not merely the presence of contextual signaling that augments learning performance in our model, but the specific astrocyte-dependent dynamical mechanisms by which this information alters neurons and synapses.

Following the above, we emphasize that our goal was not simply to add slow modulation to neuronal networks, since this could be done in a myriad of ways. Rather we chose a specific, biologically motivated and hypothesized pathway (involving astrocytes). While we cannot exclude the role of all other slow processes, we do believe that the astrocyte meta-plasticity pathway has unique advantages. To illustrate this, we pose a simple null model where we eliminated astrocytes but added a slow, passive potassium gating to neurons (see Section F.7 in S1 Appendix). This null model enacts a slow time-scale, but without the nested feedback loop structure that we believe is key to neural-astrocyte interaction. After the same training procedure as in our primary results, we find that this null model can only learn the stationary (but not the non-stationary) version of the task (see Section F.7 in S1 Appendix). This lends credence to the idea that the unique interactivity properties of astrocytes with neurons are important to the hypothesized functional benefits.

### Consistency with biology of spatiotemporal neuron-astrocyte interactions

In this paper, we have focused most of our attention on the temporal separation of neuronal and astrocytic activity and have discussed the consistency of our parameters with known biophysics in this regard. However, equally interesting are spatial features such as the ratio of astrocytes to neurons. In biology, the ratio of astrocytes to neurons is believed to be between 1:1.5 to 1:2 and we used the latter end of this range in our results. However, we also performed a sensitivity analysis to examine whether dynamics change appreciably as a function of this ratio. In the stationary case, an increased ratio positively influences learning performance, leading to a reduction in asymptotic cumulative regret as more astrocytes are introduced. However, the dynamic nature of the flip-flop bandit introduces a subtle impact on the ratio: optimal cumulative regret occurs at *intermediate* ratios, whereas both excessively low and high ratios detrimentally affect learning by increasing regret (see Section F.6 in S1 Appendix). It turns out that the optimal ratio is around 7/10, hence our simulation observation is consistent with the biology and indeed predicts a functional optimum within this range.

### Astrocytic activity as a stabilizer of catastrophic forgetting

Catastrophic forgetting is a phenomenon in artificial neural networks that arises when networks are tasked with learning multiple tasks sequentially [63]. In this scenario, it often is the case that previously encountered tasks are ‘overwritten’ when the algorithmic optimization (i.e., learning) strategies are deployed to update the network parameters/weights to meet new task demands. Our results indicate that astrocytic modulation of neuronal and synaptic dynamics mitigates catastrophic forgetting. Here, we believe that the slow time-scale of astrocytes is instrumental in protecting previously learned network outputs upon encountering of a new context. As described above, the slow activation of astrocytes makes them generally less sensitive to parametric adjustment relative to neurons and synapses. Thus, their effects are more stable context-to-context. Furthermore, as we have seen, astrocytes have the effect of controlling neuronal and synaptic dynamics, such that those faster processes can occupy distinct regions of state space depending on astrocytic modulation. The combination of these two phenomena means that astrocytes can effectively insulate the learned trajectories/dynamics of one context preventing overwriting when learning is engaged for a subsequent context. These findings underscore the importance of dynamical heterogeneity in the brain and support the functional advantages that astrocytes may confer.

Clearly, an important next step for these models will be to validate them with appropriate experiments. As mentioned in the Introductions, tools for *in vivo* study of astrocyte function have lagged relative to those for neurons. For those tools that do exist, e.g., to disrupt astrocyte function [64], studies such as ours can identify salient behavioral paradigms within which experiments may be conducted. Specifically, our model suggests that astrocytes contribute to learning in context-dependent or, potentially, multi-task settings, more than they might in simpler behavioral paradigms. This can be tested using adequate history-dependent learning tasks and popular astrocyte silencing tools such as CalEx [65]. Hence, available tools could be deployed to test formally the role of astrocytes in such scenarios. For example, by examining the learning efficacy of rodents engaging multi-arm bandit paradigms [66].

### Insights into algorithmic learning systems

While our goal in this paper has been to explore new theories regarding the potential significance of neuron-astrocyte interactions in the brain, it is nonetheless interesting to consider the implications of these results in the domain of algorithmic systems. We have already commented on the fact that traditional algorithmic methods of learning bandit tasks have difficulty in context-dependent settings, even in the presence of informative signaling. This begs the question of whether neuron-astrocyte type architectures may have utility beyond the bandit/reinforcement learning settings.

In this regard, there certainly exist recurrent neural networks designed to deal with multiple time-scale features, notably LSTMs [67] and hierarchical RNNs [68]. The LSTM has an internal memory cell state that enables it to deal with tasks that involve long-term dependencies. In hierarchical RNNs, multiple layers of RNNs are stacked on top of each other, where each layer captures information at a different level of temporal abstraction. The lower layers focus on short-term dependencies, while the higher layers focus on longer-term dependencies. The multi-scale neuron-astrocyte network considered here is in the form of a feedback-connected multi-layered network with different embedded time-scales, and hence may blend the different features of these extant machine learning architectures. It is thus possible that this framework may be extendable to other machine learning domains, especially ones involving disparate time scale requirements such as meta-learning [69].

### Limitations and features not explained

Our model suggests a key role of slow astrocytic modulation of synaptic plasticity in enabling learning over long time-scales. This model effect was premised on prior theory and empirical findings, including [14]. However, astrocyte interactions with synapses are heterogeneous across and within brain regions (see [70] for instance) both in extent (number of synapses impinged upon) and nature (pre-, postsynaptic, or both). Hence the effects we show in this paper should be interpreted as a demonstration of sufficiency rather than necessity, and certainly not monolithically across brain areas. Importantly, we do not imply that all slow brain dynamics are enacted by astrocytes. Indeed, recent evidence indicates that slow oscillatory activity in the entorhinal cortex may enable information processing across minutes or longer [71]. Our findings demonstrate that: (i) astrocytes are particularly apt to convey active, calcium-mediated modulation of synaptic dynamics, and (ii) this modulation is particularly potent in learning scenarios, relative to more diffuse and passive slow dynamics (e.g., potassium, as discussed previously above). As also pointed out above, these results set up clear potential for future empirical work aimed at the disruption of astrocytic calcium signaling in complex function.

Other important facets of astrocytes are their gap junction coupling, which is believed to be a basis for a form of functional inter-connectivity, as well as their structured arrangement in non-overlapping domains throughout the entire brain, something referred to as tiling [72]. While our model described nested feedback loops of astrocyte-neuron modulation (i.e., connectivity), we did not explicitly explore the role of tiling and gap-junction couplings, leaving this question as future work.

## Methods

### Multi-scale modeling of neuron-astrocyte network dynamics

In general, neural dynamics can be described by recurrent neural network models. Here, we consider the biology-inspired continuous-time RNN (CTRNN) [57, 73]. Consider a group of *n* neurons where each neuron is connected to some other neurons via synapses. Let $x_{i}\in R$ be the state of the unit *i*, which denotes the mean membrane potential of the neuron. Then, the model of CTRNN is defined by ODEs

$$\begin{array}{c} \tau_{n}{\dot{x}}_{i}=-a_{i}x_{i}+\sum_{j=1}^{n}w_{ij}\phi \left(x_{j}\right)+u_{i},\;i=1,\ldots ,n, \end{array}$$ (4)

where *τ*_*n*_ > 0 and *a*_*i*_ > 0 are the time constant and decaying parameter respectively, and *u*_*i*_ is the external input to unit *i*. *ϕ*(*x*_*j*_) is the activation function. It is noted that each unit *i* collects the outputs *ϕ*(*x*_*j*_) (i.e., short-term average firing frequency) from all the connected neural units in the network, weighted with the synaptic connection coefficients $w_{ij}\in R$, where the positive or negative *w*_*ij*_ indicates an excitatory or inhibitory synapse respectively.

Synapses are capable of modifying their strength via synaptic plasticity, which is usually formulated as a learning rule where the change of a synaptic strength *w*_*ij*_ depends on the correlation between the firing rate of a presynaptic neuron *j* and the firing rate of the postsynaptic neuron *i*. We consider the Hebbian learning rule: the weight between two neurons strengthens when they are correlated and weakens otherwise. This rule is defined mathematically by the equation [74]

$$\begin{array}{c} \tau_{w}{\dot{w}}_{ij}=-b_{ij}w_{ij}+c_{ij}\phi \left(x_{i}\right)\phi \left(x_{j}\right), \end{array}$$ (5)

where *b*_*ij*_ > 0 is the decaying parameter; *τ*_*w*_ > 0 is the time constant; $c_{ij}\in R$ is a parameter which indicates an existing synaptic connection when it is non-zero. When *c*_*ij*_ takes a positive value, (5) is called the *Hebbian learning*, and the case with *c*_*ij*_ < 0 is *anti-Hebbian learning*.

In principle, neurons influence astrocytes by releasing neurotransmitters that induce calcium ion elevations within astrocytes. Biophysically, the increase in calcium levels within individual astrocytes can propagate to neighboring astrocytes over long distances, forming calcium waves [75]. The mechanisms of this propagation may involve astrocyte to astrocyte gap junctions, which are well validated biologically [59, 76] and are believed to form spatially contiguous groups of astrocytes referred to as a network [77] (but also see astrocyte syncytium [78]). Current biophysical mathematical models for astrocytes, including gap junction connectivity, are excessively complex and not easily translatable for analytical and computational purposes. In the development that follows, we propose a simplified model to describe astrocyte dynamics based on [79], which abstracts the mathematical description of astrocyte-to-astrocyte connectivity within a network formulation.

Consider a group of *m* astrocytes. Let $z_{k}\in R$ be the state of astrocyte *k* which denotes the activity of calcium wave. For the glial node *z*_*k*_, we assume the output of astrocyte calcium wave is similarly defined by an activation function. To distinguish it from the neuron, we use a different function, for instance, the hyperbolic tangent function *ψ*(*z*_*k*_) = tanh(*z*_*k*_). Then, in the absence of neuron-astrocyte interactions, the dynamics of *z*_*k*_ is described by

$$\begin{array}{c} \tau_{a}{\dot{z}}_{k}=-e_{k}z_{k}+\sum_{l=1}^{m}f_{kl}\psi \left(z_{l}\right)+v_{k},\;k=1,\ldots ,m, \end{array}$$ (6)

where *τ*_*a*_ is a constant time parameter; *f*_*kl*_ denotes the weight of the (network) connection from astrocyte *l* to *k*; *v*_*k*_ captures other external inputs. The usage of this phenomenological model can be justified with analogous arguments in [34], where a neuronal leaky integrate-and-fire model is used for astrocytes. Such a model is easy to modify to incorporate the neuro-synapse-astrocyte interactions and greatly facilitates the numerical and analytical investigation as shown in the first subsection of Results.

Stacking all the equations of neurons, synapses, and astrocytes together, we will arrive at the mathematical model for the neuron-astrocyte network as a whole.

$$\begin{array}{cc} & \tau_{n}{\dot{x}}_{i}=-a_{i}x_{i}+\sum_{j=1}^{n}w_{ij}\phi \left(x_{j}\right)+u_{i},\;i=1,\ldots ,n, \end{array}$$ (7a)

$$\begin{array}{cc} & \tau_{w}{\dot{w}}_{ij}=-b_{ij}w_{ij}+c_{ij}\phi \left(x_{i}\right)\phi \left(x_{j}\right)+d_{ij}\psi \left(z_{k}\right),\;i,j=1,\ldots ,n, \end{array}$$ (7b)

$$\begin{array}{cc} & \tau_{a}{\dot{z}}_{k}=-e_{k}z_{k}+\sum_{l=1}^{m}\;f_{kl}\psi \left(z_{l}\right)+h_{k}\phi \left(x_{i}\right)\phi \left(x_{j}\right)+v_{k},\;k=1,\ldots ,m, \end{array}$$ (7c)

where the additional terms *d*_*ij*_*ψ*(*z*_*k*_) and *h*_*k*_*ϕ*(*x*_*i*_)*ϕ*(*x*_*j*_) with $d_{ij},h_{k}\in R$ are present to capture the high-order interaction between neurons, astrocytes and synapses according to the description in tripartite synapse structure. In system (7), there are *n* and *m* equations for *x* and *z* respectively. The number of synaptic connections is flexible and denoted by *o* with *m* ≤ *o* ≤ *n*(*n* − 1). Therefore, the dimension of system (7) is actually (*m* + *n* + *o*).

It is known that the activities of neurons, synapses, and astrocytes evolve on different time-scales. Neural firing occurs in milliseconds, synapse plasticity changes at a slower speed, and astrocyte processes take even longer, ranging from seconds to minutes. These varying time-scales significantly impact information processing in neuron-astrocyte interactions. To investigate the effects of these differences, we need to set the time-scale parameters, denoted as *τ*_*n*_, *τ*_*w*_, and *τ*_*a*_, to different values. To make the speeds of the evolution of these variables distinguishable, we have the assumption: 0 < *τ*_*n*_ ≪ *τ*_*w*_ ≪ *τ*_*a*_, with ≪ indicating the former entity is much smaller than the latter. As the main goal of this work is to study neuron and astrocyte computation, we set *τ*_*n*_ = *τ*_*w*_ for simplicity when applying the neuron-astrocyte model to solving the tasks.

### Dynamic context-dependent multi-armed bandit tasks

In the setting of a stochastic MAB, there is a set of actions (arms) A to choose from, and the bandit lasts *T* rounds in total. In each round *t*, an agent (decision-maker) chooses one action $a_{t}\in A$ and obtains a reward *r*_*t*_. The goal of the agent is to optimize the accumulated reward, i.e., ${\text{max}}_{a_{t}\in A}\;\sum_{t=1}^{T}r_{t}$. We consider the Bernoulli bandits which belong to stochastic MABs. In the context of Bernoulli bandits, the reward of each action is binary, either 1 or 0 depending the outcome is a success or failure. The reward *r*_*i*_ of the *i*-th action is drawn from a Bernoulli distribution, i.e.,

$$r_{i}∼\text{Bernoulli}\left(\mu_{i}\right),\;\;i=1,\ldots ,n,$$

where *μ*_*i*_ ∈ [0, 1] is a constant denoting the mean of the distribution. Different actions have different *μ*_*i*_ where a larger value represents a higher probability of the successful outcome and thus a higher expectation of the reward. The reward sequence up to time *T* is a random process

$$\begin{array}{c} \left\{r_{t}∼{\left\{\text{Bernoulli}\left(\mu_{i}\right)\right\}}_{i=1}^{n},\;\;t=1,\ldots ,T.\right\} \end{array}$$ (8)

In the Bernoulli bandit, the goal of optimizing the accumulated reward is equivalent to minimizing the cumulative regret (3). The standard Bernoulli bandit is stationary where all *μ*_*i*_ are fixed over time. In addition to the stationary case, we further consider non-stationary variants by making the means changeable and time-dependent. Two subcases are considered in this work:

1. Flip-flop switching: the means *μ*_*i*_ of actions remain constant for a certain period of time, and then abruptly transit to different values $\mu_{i}^{\prime }\in \left[0,1\right]$ at certain time instants.
2. Smooth changing: the means change according to a continuous function of time. Here, we use the periodic function
  $$\begin{array}{c} \mu_{i}\left(t\right)=\mu_{i}^{*}S(Q\;\text{sin}\;(\frac{2\pi t}{P}+\frac{2\pi i}{n})), \end{array}$$ (9)
  where $\mu_{i}^{*}$ is a fixed value in [0, 1]; *S*(⋅) is the sigmoid function; *P* is used to control the period of this function and the term $\frac{2\pi i}{n}$ makes that the action with the highest expected reward can change between the available actions over time. When *Q* is large, this type of function is dominated by an approximately constant value, such that it looks like a smooth square wave. We set *P* and *Q* to 10000 and 100 respectively.

In dynamic bandits, when the arm means change over time and the action with the highest mean switches, contextual information can be revealed to the agent. This contextual information represents the changes in underlying contexts. Therefore, the tasks we considered become context-dependent. We define the contextual signals as a scalar in all the simulations presented in this work. However, it is important to note that these signals can also be expanded into a multi-dimensional vector to accommodate more general settings.

### Discrete-time neuron-astrocyte network

For simplification, we assume that the self-decay parameters are all one and the time-scales of neurons and synapses are the same. Then, the neuron-astrocyte network model without inputs can be rewritten in the compact form

$$\begin{array}{c} \tau \dot{x}=-x+W\phi \left(x\right)\\ \tau \dot{W}=-W+C\Phi \left(x\right)+D\psi \left(z\right)\\ \dot{z}=-z+F\psi \left(z\right)+H\Phi \left(x\right), \end{array}$$ (10)

where *x* = [*x*_1_, …, *x*_*n*_]^⊤^ and *z* = [*z*_1_, …, *z*_*m*_]^⊤^ are state vectors for neurons and astrocytes; *W* = [*w*_*ij*_] is the matrix for synapse weights and $\dot{W}$ denotes the element-wise derivative of *W*; *ϕ*(*x*) = [*ϕ*(*x*_1_), …, *ϕ*(*x*_*n*_)]^⊤^ and *ψ*(*z*) = [*ψ*(*z*_1_), …, *ψ*(*z*_*m*_)]^⊤^ are vectors of activation functions while Φ(*x*) is the flatten vector of the matrix [*ϕ*(*x*_*i*_)*ϕ*(*x*_*j*_)]; *C*, *D*, *F*, and *H* are the parameter matrices with corresponding entries.

In (10), we have set the time constant for astrocytes to the unit, while time constants for neurons and synapses are both *τ* ≪ 1. In this way, *τ* is dimensionless and represents the time-scale difference rate between neurons and astrocytes. Note that (10) can be rewritten equivalently by a change of time, so that *τ* appears on the right hand side of $\dot{z}$.

By using the first-order Euler discretization method [80], we can transfer the continuous-time neuron-astrocyte model to the discrete-time approximated version

$$\begin{array}{c} x_{t}=\left(1-\gamma \right)x_{t-1}+\gamma W_{t-1}\phi \left(x_{t-1}\right)\\ W_{t}=\left(1-\gamma \right)W_{t-1}+\gamma \left(C\Phi \left(x_{t-1}\right)+D\psi \left(z_{t-1}\right)\right)\\ z_{t}=\left(1-\gamma \tau \right)z_{t-1}+\gamma \tau \left(F\psi \left(z_{t-1}\right)+H\Phi \left(x_{t-1}\right)\right), \end{array}$$ (11)

where *γ* is the discretization step size. In the following simulations, *γ* and *τ* are set to be 0.1 and 0.01 respectively. We use the sigmoid function *ϕ*(*x*) = 1/(1 + *e*^−*x*^) and the hyperbolic tangent function *ψ*(*z*) = tanh(*z*) for neural and astrocyte layer in the simulations.

We incorporate this discrete-time neuron-astrocyte model as the hidden layer within the entire learning network, where a pair of linear input and output layers are placed before and after the hidden layer according to the machine learning convention. The input $I\in R^{\left|u\right|}$ and the output $y\in R^{\left|y\right|}$ are feed into and read from neuron-astrocyte network after multiplied by matrices $W_{\text{in}}^{1},W_{\text{in}}^{2}$ and *W*_out_. Therefore, the network as a whole is represented by

$$\begin{array}{c} x_{t}=\left(1-\gamma \right)x_{t-1}+\gamma \left(W_{t-1}\phi \left(x_{t-1}\right)+W_{\text{in}}^{1}I_{t}\right)\\ W_{t}=\left(1-\gamma \right)W_{t-1}+\gamma \left(C\Phi \left(x_{t-1}\right)+D\psi \left(z_{t-1}\right)\right)\\ z_{t}=\left(1-\gamma \tau \right)z_{t-1}+\gamma \tau \left(F\psi \left(z_{t-1}\right)+H\Phi \left(x_{t-1}\right)+W_{\text{in}}^{2}I_{t}\right)\\ y_{t}=W_{\text{out}}x_{t}+b_{\text{out}}, \end{array}$$ (12)

where *b*_out_ the bias vector with the corresponding dimension.

### Reinforcement learning procedure

A key step of our study is the implementation of our model to reinforcement-learning paradigms. Within this functional setting, at each trial, the agent (i.e., the neuron-astrocyte network) is presented with a new reward. This reward is used to algorithmically optimize (i.e., train) the parameters of the model in a trial-wise fashion. In other words, at the conclusion of the trial, the current parameters and outputs of the model, along with the current reward, are used to evaluate a loss function (see below) that determines future parameter adjustments. The research question at hand is whether the neuron-astrocyte architecture and dynamics enable this form of learning to be efficacious. Table 1 summarizes all parameters, both fixed and trainable, and their values in the model and training process.

**Table 1:** Parameters in the neuron-astrocyte model and model training.

Symbols	Description	Values
*n*	number of neurons	128
*m*	number of astrocytes	64
*τ*	time-scale parameter	0.01
*γ*	discretization step	0.1
*I*	contextual cues	stationary case: {1} flip-flop: {−1, 1} smooth: {−1, 0, 1}
$W_{in}^{1},W_{in}^{2}$	input weight matrices	Trained
*C*, *D*	matrices associated with synapses	Trained
*F*, *H*	matrices associated with astrocytes	Trained
*W*_*out*_	output weight matrix	Trained
*b*_*out*_	output bias vector	Trained

The neuron-astrocyte network architecture comprises 128 neurons and 64 astrocytes (except for simulations where we vary the neuron-astrocyte ratio), with randomly initialized connections within each layer and interlayer hyperedges. The complete learning framework is depicted in Fig 3A. We first initialize the matrices *C*, *D*, *F*, *H* with entries drawn randomly from normal distributions with the zero mean, i.e.,

$$M_{ij}∼\frac{1}{\sqrt{N_{M}}}N\left(0,1\right),$$

where *N*_*M*_ is the dimension of the focal matrix *M*. The elements of input and output matrices $W_{\text{in}}^{1}$, $W_{\text{in}}^{2}$, *W*_out_ and bias vector *b*_out_ are initialized from a uniform distribution $U(-\frac{1}{\sqrt{N_{M}}},\frac{1}{\sqrt{N_{M}}})$, where *N*_*M*_ is again the dimension.

The dimension of the output *y*_*t*_ is the same as the number of actions in the bandits, i.e., 3 in most simulations. After multiplied by the readout matrix and plus the bias, the output is fed to a softmax function, and it produces a probability distribution over the available actions $p_{t}=\left[p_{t}^{1},p_{t}^{2},p_{t}^{3}\right]$. The probability of selecting the action $a_{i}\in A$ is

$$\begin{array}{c} p_{t}^{i}=\frac{e^{y_{i}}}{\sum_{1}^{3}\;e^{y_{j}}},i=1,2,3. \end{array}$$ (13)

An action *a*_*t*_ is then sampled from this probability distribution and subsequently executed by the agent. The bandit environment will provide the agent with a reward, represented as $r_{a_{t}}$. And according to [81], we use the loss function

$$L=\left({\bar{r}}_{t}-r_{a_{t}}\right)\;\text{log}\;p_{t}^{i},$$

where ${\bar{r}}_{t}$ is the average of rewards up to *t* and $\text{log}\;p_{t}^{i}$ is the logarithm of the probability.

We adopted the traditional policy-based RL algorithm *REINFORCE* for the network training [81]. The gradient of the loss function *L* is calculated and used to update the network’s free parameters via the backpropagation (BP). During BP, we use the Adam method to optimize the aforementioned matrices and vectors with the default learning rate of 0.001.

In the case of other RNN-based methods as described in the comparison section below, we simply replace the neuron-astrocyte network with the alternative network models. To ensure a fair comparison of a comparable magnitude of training parameters, all these conventional RNNs are configured with 2 stacked layers, each consisting of 128 units. In the 2 stacked layers structure, the first layer is forward-connected to the second layer: the external input (contextual cues) is fed to the first layer, which has default trainable intra-connection weights; the output of the first layer is fed to the second layer as the input associated with a trainable matrix; and then the output of the second layer is further used to generate actions. The weights are initialized using a default method, and the training procedure remains consistent.

The network models and training procedures are implemented using PyTorch in Python.

### Learning performance comparison

Numerous machine learning algorithms have been developed to tackle MABs. Among them, Upper Confidence Bound (UCB) and Thompson Sampling (TS) are widely recognized as the most prominent approaches for standard MABs. Discounted UCB (DUCB) and switching-window UCB (SWUCB) have been devised to handle changing environments in non-stationary scenarios. In addition to these canonical bandit algorithms, some neuro-bandit algorithms that utilize feedforward or recurrent neural networks to model the agent’s policy have been developed in recent years.

To perform a thorough yet not overly exhaustive assessment of learning performance, we analyze the asymptotic cumulative regret of our approach in comparison to selective algorithms across various scenarios. For stationary MABs, we evaluate our method against the UCB and TS algorithms, as well as RNN-based models including LSTM, vRNN, and GRU. In the context of non-stationary MABs, our method is compared to DUCB, SWUCB, and other RNN-based algorithms. It’s worth noting that the training procedures for all RNN-based models remain consistent with the previously described methodology.

## Supporting information

S1 Appendix The supplementary appendix file contains the mathematical analysis of the models and extensive simulation results stated in the main text. (PDF)
