# Supplementary material for: Astrocytes as a mechanism for contextually-guided network dynamics and function
Source: PLoS Comput Biol. 2024 May 31;20(5):e1012186. doi: 10.1371/journal.pcbi.1012186 (PMC11168681; doi:10.1371/journal.pcbi.1012186)
Supplement: S1 Appendix — (PDF) [file pcbi.1012186.s001.pdf]

Appendix of "Astrocytes as a mechanism for contextually-guided network dynamics and function"

Lulu Gong*, Fabio Pasqualetti, Thomas Papouin, and ShiNung Ching

A Enhanced understanding of signal flow in the tripartite synapse

As stated in the main text, we can reveal the signal flow in the tripartite synapse more apparently via the symbolic description. We denote the activities associated with the neurons and the astrocyte by symbols V_N^1, V_N^2, and V_A respectively, and the synaptic efficacy is V_S. Then, the interplay between astrocytes and neuronal elements can be represented by different arrows as shown in Fig 1, where two instrumental feedback-loops are identified in the flow. In the first loop, the signal flows from Neuron 1 to Neuron 2 through the synapse, and Neurons 1 and 2's signals act on the synapse's efficacy (so-called Hebbian plasticity); Meanwhile, Neurons 1 and 2's signals also affect the astrocyte's activity and in turn acts on the synaptic efficacy, and thus form the second loop based on the signal flow from Neuron 1 to Neuron 2. It is noticed that the neurons 1 and 2's signals together act on the synapse and astrocyte. This integration of two signals is drastically different from two separated signals and thus forms a high-order interaction.

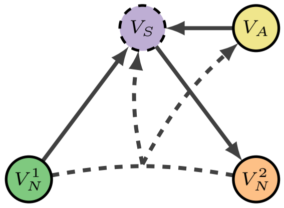

Figure 1: The signal flow in the tripartite synapse structure.

B Graphical description of neuron-astrocyte population as a hypernetwork

We have claimed that the neuron-astrocyte population can be described by a two-layer hypernetwork, and now we will introduce a formal graphical description of this hypernetwork.

The brain is usually described as a network where nodes represent neurons and (directed) edges between nodes denote synaptic connections, which can be inhibitory or excitatory. In network theory, a generic (mono-layer) network can be defined by a graph $G := (N, E)$, where $N = \{1, ..., n\}$ is the set of nodes; $E = \{(i, j) |\ i, j \in N \text{ and are connected}\}$ is the set of edges. We consider the directional and weighted graph. That means an edge in E, e.g., (i, j), has the direction from node i to node j and owns a weight $w_{ij} \in \mathbb{R}$. In addition, a network can have multiple layers (neural and astrocytic) that can have the same or different nodes [1]. When considering a large number of neurons and astrocytes presenting in the brain system, we need to separate neurons and astrocytes as two groups because of their natural differences. In this regard, we prescribe that the neuron-astrocyte populations have two different layers, with each layer representing the group of neurons and astrocytes respectively. And they are denoted by the graphs $G_n = \{N_n, E_n\}$ and $G_a = \{N_a, E_a\}$ respectively.

On the other hand, an edge only connects two nodes and thus describes the pairwise interaction. Because of the presence of high-order interaction within the tripartite synapse, the interconnections between the two layers cannot be represented by normal edges. We then extend it to hyperedges that can connect any number of nodes. A hyperedge H_i is defined as a subset of N satisfying $H_i \neq \emptyset$.

Then, the whole neuron-astrocyte network can be denoted by the hypernetwork $\mathcal{M} = \{G_n, G_a, \{H_i\}\}$. This hypernetwork contains two layers, i.e, the neural layer and the astrocytic layer. The neural layer consists of all the neurons and the intralayer network structure is consistent with the standard directional neural network; the astrocytic layer includes the astrocytes and it embeds the directional network structure as well. Edges that connect a node to itself (self-loops) are excluded for both layers. Suppose there are neurons interacting with an astrocyte. In that case, an interlayer connection takes place therein, and we use a hyperedge to represent it: each hyperedge (the triangle shape in Fig 1B of Main Text) connects one astrocyte and two neurons. Noting that the hyperedge essentially includes the edges connecting the two neurons which represent the plastic synapses. In this sense, the defined hyperedge can well capture the high-order interactions be-

tween two neurons, the astrocyte and synapse. As a result, the neuron-astrocyte ensemble structure is well represented by this two-layer hypernetwork.

C Well-definiteness of the neuron-astrocyte network model

From the mathematical point of view, it is important to check if the neuron-astrocyte network model is posed and defined correctly. Our model is given by a set of continuous-time ODEs. For such a system, it is well-posed if the solution to an initial value problem exists and is unique. The well-posedness is guaranteed if the vector field of the model is Lipschitz continuous in the variables and continuous in time [2]. It is well-known that common activation functions, such as the logistic sigmoid and $tanh$ are Lipshitz. In the presumption that the external inputs are continuous in time, our model is well-posed.

The model is also well-defined in the sense that the dynamics will not expand without restriction but will be confined in an appropriate subspace in the real space for any initial conditions after some certain time as shown in Section 4. Moreover, we can approximately estimate this region using mathematical arguments as shown in the following.

D Normal analysis of network motif dynamics

As the neuron-astrocyte model in this work is proposed for the first time, it is necessary to conduct a normal analysis including studying the boundedness and fixed points conditions. The network is composed of network motifs, it is enough to consider the minimal model on the network motifs.

Boundedness Note the network motif dynamics are given by

$$\begin{aligned} \tau_1 \dot{x}_1 &= -a_1 x_1 + w_2 \phi(x_2) + u_1(t) \\ \tau_1 \dot{x}_2 &= -a_2 x_2 + w_1 \phi(x_1) + u_2(t) \\ \tau_2 \dot{w}_1 &= -b_1 w_1 + c_1 \phi(x_1)\phi(x_2) + d_1 \psi(z) \\ \tau_2 \dot{w}_2 &= -b_2 w_2 + c_2 \phi(x_1)\phi(x_2) + d_2 \psi(z) \\ \tau_3 \dot{z} &= -ez + h\phi(x_1)\phi(x_2) + v(t). \end{aligned} \tag{1}$$

We first define the boundedness of a general dynamical system without inputs.

Definition 1. *Given a dynamical system*

$$\dot{x} = f(x),$$

where $f : \mathbb{R}^n \to \mathbb{R}^n$ *is continuous. Let* $x(t),\ t \geq 0$ *be a trajectory of the system.* $x(t)$ *is said to be* ultimately bounded *if there exist* $M > 0$ *and* $T > 0$ *such that* $\|x(t)\| \leq M$ *for all* $t \geq T$*. Moreover, the system is said to be ultimately bounded if all trajectories are ultimately bounded.*

For system (1) without external inputs, although the vector field are defined for $\mathbb{R}^5$, we can show that the dynamics are indeed bounded after some certain time. Let $X = (x_1, x_2, x_3, w_1, w_2, z)^\top$. As the activation functions are bounded, we denote the maximum values of $\phi(\cdot)$ and $\psi(\cdot)$ by $M_1 > 0$ and $M_2 > 0$ respectively. Define the set

$$\Omega := \left\{ X \in \mathbb{R}^5 : \begin{array}{c} |x_1|, |x_2| \leq x_{\max} \\ |w_1|, |w_2| \leq w_{\max} \\ |z| \leq z_{\max} \end{array} \right\},$$

with $x_{\max} = w_{\max} M_1 / \min\{a_1, a_2\}$,
$w_{\max} = (\max\{|c_1|, |c_2|\} M_1^2 + \max\{|d_1|, |d_2|\} M_2 / \min\{b_1, b_2\}$ and $z_{\max} = |h| M_1^2 / e$.

Theorem 1. *In the absence of external inputs, the dynamics of* (1) *are ultimately bounded in the set* Ω.

Proof: Let $\bar{z}(t)$ be the solution of the differential equation

$$\dot{\bar{z}} = -e\bar{z} + |h| M_1^2, \ \ \bar{z}(0) = z(0).$$

One can obtain that $\bar{z}(t) = (z(0) - |h| M_1^2 / e) \exp(-et) + |h| M_1^2 / e$, which yeilds

$$|\bar{z}(t)| \leq |(z(0) - |h| M_1^2 / e) \exp(-et)| + h |M_1^2 / e.$$

As $e > 0$, the first term on the right hand side of the above inequality converges to zero exponentially. Therefore, there exist a time $T > 0$ such that $|\bar{z}(t)| \leq h |M_1^2 / e$ for all $t > T$. On the other hand, from the last equation of (1), we have $\dot{z} \leq \dot{\bar{z}}$. Then, by the comparison lemma [3, Lemma 3.4], we get $|z(t)| \leq |\bar{z}(t)|$. It follows that there exists a time T_1 such that $|z(t)| \leq h |M_1^2 / e$ for all $t > T_1$.

Analogously, we can derive the bound for the other variables as defined in Ω. Finally, we prove that the dynamics of (1) are ultimately bounded in the set Ω.

Along with the Theorem of boundedness, we have some remarks.

Remark 1. *1. Here, we focus on the autonomous system. With the proper condition that the inputs are bounded, one can show the boundedness of the system when external inputs are presented [4].*

2. The set Ω is positive invariant and attractive with respect to (1). *Intuitively, by the definition of limit points [5], all the positive limit points of system* (1)*, such as fixed points and limit cycles, must be included in the attractive set Ω. This property helps obtain the following results about fixed points.*

Fixed points With the boundedness in hand, we can study the fixed points of the system. Continue considering the system (1) without external inputs. Letting the right hand sides of (1) be zero results in the following equations

$$\begin{aligned} x_1 &= \frac{w_2\phi(x_2)}{a_1} \\ x_2 &= \frac{w_1\phi(x_1)}{a_2} \\ w_1 &= \frac{c_1\phi(x_1)\phi(x_2) + d_1\psi(z)}{b_1} \\ w_2 &= \frac{c_2\phi(x_1)\phi(x_2) + d_2\psi(z)}{b_2} \\ z &= \frac{h\phi(x_1)\phi(x_2)}{e}. \end{aligned} \tag{2}$$

Each of the above equations defines a nullcline in $\mathbb{R}^5$, and together their solutions (intersections of nullclines) yield the fixed points.

First, let us consider the existence of fixed points, which can be proved easily by using the Brouwer's fixed point theorem [6]. Define the mapping

$$F := \begin{pmatrix} \frac{w_2\phi(x_2)}{a_1} \\ \frac{w_1\phi(x_1)}{a_2} \\ \frac{c_1\phi(x_1)\phi(x_2) + d_1\psi(z)}{b_1} \\ \frac{c_2\phi(x_1)\phi(x_2) + d_2\psi(z)}{b_2} \\ \frac{h\phi(x_1)\phi(x_2)}{e} \end{pmatrix}.$$

Theorem 2. *System* (1) *has (at least) a fixed point in* Ω.

Proof: It is easy to check that the defined mapping F is continuous. To prove the existence of fixed points, according to Brouwer's Fixed-point Theorem, we only need to show the set Ω is compact and convex. Since Ω is bounded and closed, the compactness follows. In addition, the set Ω actually defines a hyper rectangle in $\mathbb{R}^5$, which is convex. Therefore, we can conclude that there exists (at least) one fixed point of system (1) in Ω.

Next, we examine the uniqueness of the fixed point in (1). The Jacobian of F is given by

$$DF = \begin{bmatrix} 0 & \frac{w_2\phi'(x_2)}{a_1} & 0 & \frac{\phi(x_2)}{a_1} & 0 \\ \frac{w_1\phi'(x_1)}{a_2} & 0 & \frac{\phi(x_1)}{a_2} & 0 & 0 \\ \frac{c_1\phi'(x_1)\phi(x_2)}{b_1} & \frac{c_1\phi(x_1)\phi'(x_2)}{b_1} & 0 & 0 & \frac{d_1\psi'(z)}{b_1} \\ \frac{c_2\phi'(x_1)\phi(x_2)}{b_2} & \frac{c_2\phi(x_1)\phi'(x_2)}{b_2} & 0 & 0 & \frac{d_2\psi'(z)}{b_2} \\ \frac{h\phi'(x_1)\phi(x_2)}{e} & \frac{h\phi(x_1)\phi'(x_2)}{e} & 0 & 0 & 0 \end{bmatrix} \tag{3}$$

To ensure that Eqs. (2) have a unique solution in the previously obtained set Ω, one sufficient condition is that the inequality

$$\|DF\|_\Omega = \sup_{X\in\Omega}\|DF(X)\| < 1 \tag{4}$$

holds, where $\|\cdot\|$ denotes the matrix norm. We take the 1-norm of DF, i.e., the maximum of the absolute values sum of the rows

$$\begin{aligned}\|DF(X)\| = \max\Bigg\{&\left|\frac{w_2\phi'(x_2)}{a_1}\right| + \left|\frac{c_1\phi(x_1)\phi'(x_2)}{b_1}\right| + \left|\frac{c_2\phi(x_1)\phi'(x_2)}{b_2}\right| + \left|\frac{h\phi(x_1)\phi'(x_2)}{e}\right|, \\ &\left|\frac{w_1\phi'(x_1)}{a_2}\right| + \left|\frac{c_1\phi'(x_1)\phi(x_2)}{b_1}\right| + \left|\frac{c_2\phi'(x_1)\phi(x_2)}{b_2}\right| + \left|\frac{h\phi'(x_1)\phi(x_2)}{e}\right|, \\ &\left|\frac{\phi(x_1)}{a_2}\right|, \left|\frac{\phi(x_2)}{a_1}\right|, \left|\frac{d_1\psi'(z)}{b_1}\right| + \left|\frac{d_2\psi'(z)}{b_2}\right|\Bigg\}.\end{aligned} \tag{5}$$

Note that all the variables and the derivatives of activation functions are bounded. It is always possible to find such conditions that (5) is less than 1 for all points in Ω. To showcase, let us consider the case where $\phi(\cdot) = 1/(1+e^{-x})$, $\phi'(\cdot) = e^{-x}/(1+e^{-x})^2$ and $\psi(z) = (e^z + e^{-z})/(e^z + e^{-z})$, $\psi'(z) = 1 - \psi^2(z)$. Then we have

$$\sup\left|\frac{\phi(x_1)}{a_2}\right| = \frac{1}{a_2},$$

$$\sup\left|\frac{\phi(x_2)}{a_1}\right| = \frac{1}{a_1},$$

$$\sup\left(\left|\frac{d_1\psi'(z)}{b_1}\right| + \left|\frac{d_2\psi'(z)}{b_2}\right|\right) = \left|\frac{d_1}{b_1}\right| + \left|\frac{d_2}{b_2}\right|,$$

$$\sup\left(\left|\frac{w_1\phi'(x_1)}{a_2}\right| + \left|\frac{c_1\phi'(x_1)\phi(x_2)}{b_1}\right| + \left|\frac{c_2\phi'(x_1)\phi(x_2)}{b_2}\right| + \left|\frac{h\phi'(x_1)\phi(x_2)}{e}\right|\right)$$
$$= \frac{|w_1|_{\max}}{4a_2} + \left|\frac{c_1}{4b_1}\right| + \left|\frac{c_2}{4b_2}\right| + \left|\frac{h}{4e}\right|,$$

$$\sup\left(\left|\frac{w_2\phi'(x_2)}{a_1}\right| + \left|\frac{c_1\phi(x_1)\phi'(x_2)}{b_1}\right| + \left|\frac{c_2\phi(x_1)\phi'(x_2)}{b_2}\right| + \left|\frac{h\phi(x_1)\phi'(x_2)}{e}\right|\right)$$
$$= \frac{|w_2|_{\max}}{4a_1} + \left|\frac{c_1}{4b_1}\right| + \left|\frac{c_2}{4b_2}\right| + \left|\frac{h}{4e}\right|,$$

where $|w_i|_{\max}, i = 1, 2$ are the maximum values of $|w_i|$ taking in Ω. Therefore, one has $\|DF\| < 1$ for all $X \in \Omega$ if the following conditions are satisfied

$$a_1 > 1,\ a_2 > 1,\ \left|\frac{d_1}{b_1}\right| + \left|\frac{d_2}{b_2}\right| < 1,\ \max\left\{\frac{|w_2|_{\max}}{a_1}, \frac{|w_1|_{\max}}{a_2}\right\} + \left|\frac{c_1}{b_1}\right| + \left|\frac{c_2}{b_2}\right| + \left|\frac{h}{e}\right| < 4. \tag{6}$$

Then, it follows that the mapping F is a contraction mapping in Ω [7]. A contraction mapping has the property of admitting a unique fixed point as stated in Banach's fixed point theorem [7]. According to this theorem, F has a unique fixed point in Ω , which implies the system (1) has a unique fixed point as stated in the following theorem.

Theorem 3. *When* (6) *holds, system* (1) *has a unique fixed point in the defined domain, and this fixed point is located in the set* Ω.

Remark 2. *In the above process, we used the* 1*-norm of* DF *and arrived at the sufficient conditions* (6)*. Of course, one can use other norms and thus obtain different sufficient conditions for the uniqueness of the fixed point.*

Next, to go beyond the single fixed point, we investigate the conditions for the existence of multiple fixed points. As the involvement of so many parameters and uncertain activation functions, it is difficult to fully and analytically characterize the parameter conditions for the existence of multiple fixed points. For simplicity, we restrict to the case of sigmoid and hyperbolic tangent activation functions, i.e., $\phi(x) = 1/(1 + e^{-x})$ and $\psi(z) = (e^z + e^{-z})/(e^z + e^{-z})$.

In (2), we substitute the third and fourth equations to the first two, and arrive at the following set of equations with the reduced dimension

$$\begin{aligned}
x_1 &= \frac{(c_2\phi(x_1)\phi(x_2) + d_2\psi(z))\phi(x_2)}{a_1 b_2} \\
x_2 &= \frac{(c_1\phi(x_1)\phi(x_2) + d_1\psi(z))\phi(x_1)}{a_2 b_1} \\
z &= \frac{h\phi(x_1)\phi(x_2)}{e}.
\end{aligned} \tag{7}$$

Now we presume that the values of variables x_1, x_2 and z are large so that $\phi(x_1) = 1/(1+e^{-x_1}) = 1-\sigma_1$, $\phi(x_2) = 1/(1+e^{-x_2}) = 1-\sigma_2$, and $\psi(z) = (e^z+e^{-z})/(e^z+e^{-z}) = 1-\sigma_3$ where $0 < \sigma_1, \sigma_2, \sigma_3 < 1$ are small. In doing so, (7) yields

$$\begin{aligned}
x_1^* &= \frac{c_2(1-\sigma_1)(1-\sigma_2)^2 + d_2(1-\sigma_2)(1-\sigma_3)}{a_1 b_2} \\
x_2^* &= \frac{c_1(1-\sigma_1)^2(1-\sigma_2) + d_1(1-\sigma_1)(1-\sigma_3)}{a_2 b_1} \\
z^* &= \frac{h(1-\sigma_1)(1-\sigma_2)}{e}.
\end{aligned} \tag{8}$$

To ensure that the solution given by (8) is one fixed point of system (1), the following equalities should also be satisfied

$$\begin{aligned}
&1/(1+e^{-x_1^*}) = 1-\sigma_1 \\
&1/(1+e^{-x_2^*}) = 1-\sigma_2 \\
&(e^{z^*} - e^{-z^*})/(e^{z^*} + e^{-z^*}) = 1-\sigma_3.
\end{aligned} \tag{9}$$

Now, the question turns to be finding a collection of parameters $a_1, ..., h$ such that (9) holds with $\sigma_1, \sigma_2, \sigma_3 > 0$ being very small. We further simplify this problem by assuming that $a_1 b_2 = a_2 b_1, c_1 = c_2, d_1 = d_2$ and $\sigma_1 = \sigma_2 = \sigma_3$. Then we have

$$1 + e^{-x_1^*} = 1 + e^{-x_2^*} = (e^{z^*} + e^{-z^*})/(e^{z^*} - e^{-z^*}) = 1/(1-\sigma). \tag{10}$$

Substituting (8) results in

$$\begin{aligned}
\frac{c_2(1-\sigma)^3 + d_2(1-\sigma)^2}{a_1 b_2} &= \ln\frac{\sigma}{1-\sigma} \\
\frac{2h(1-\sigma)^2}{e} &= \ln\frac{2-\sigma}{\sigma}.
\end{aligned}$$

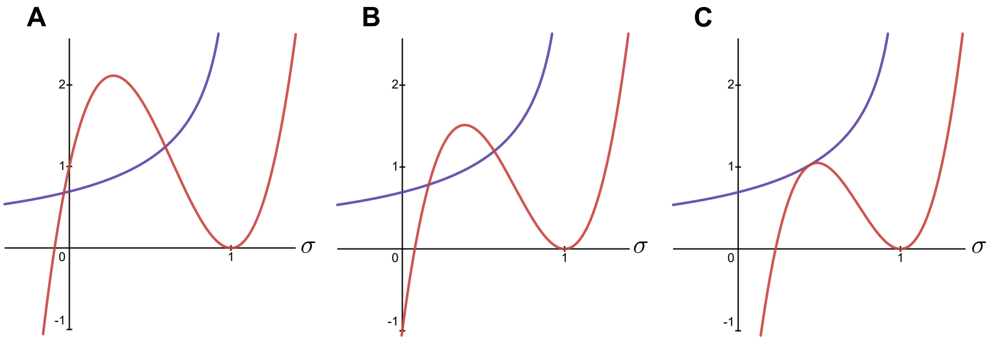

Figure 2: Intersections of two curves where the blue line is the right hand side and the brown line is the left hand side. A. one intersection when $c_2 = -1.1$; B. two intersections when $c = -1.3$; C one intersection when $c = -1.5595$.

It then yields

$$\frac{ec_2(1-\sigma)^3 + (ed_2 + 2ha_1b_2)(1-\sigma)^2}{a_1b_2e} = \ln\frac{2-\sigma}{1-\sigma} \tag{11}$$

The right hand side of (11) is in the range $\mathbb{R}_+$ and is monotonically increasing for $\sigma \in (0, 1)$. Note that a_1, b_2, c_2, d_2, e, h are free parameters. When they are all positive, the left hand side of (11) is in the range $(0, \frac{ec_2+ed_2+2ha_1b_2}{a_1b_2e})$ and is monotonically decreasing for $\sigma \in (0, 1)$. That means the two sides must have one intersection for $\sigma \in (0, 1)$. In this case, there will be a unique fixed point. On the other hand, such many free parameters can give rise to other possibilities. We can fix some parameters to be constant values, e.g., $a_1b_2 = 0.1$, $d_2 = e = 1$, $h = 1$. It can be calculated that when $-1.5595 < c_2 < -1.1307$, the two sides of (11) will always have intersections as shown in Fig 2B, which results in two fixed points for the system. It can be expected that when we release these restrictions on the parameters, it is easier for the system to have more fixed points.

Remark 3. *We have shown in the above how the system can admit two fixed points analytically and numerically. The case of multiple fixed points is not rare because of the many parameters, and an example of* 3 *fixed points can be obtained under some conditions as in Fig 2 of Main Text. To show the case of more fixed points is tedious and marginal, and thus it is out of the scope of this work.*

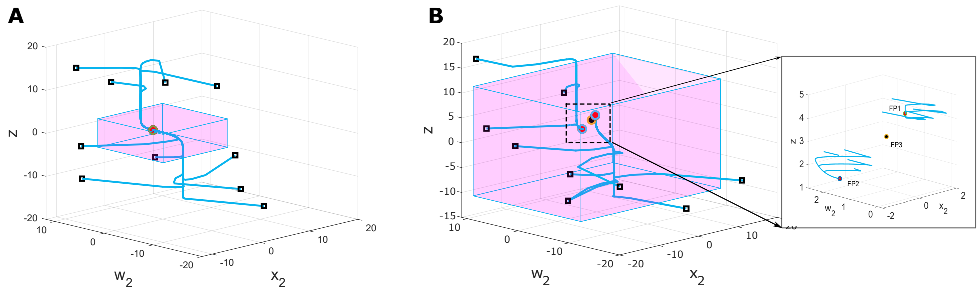

Figure 3: The cases of single and multiple fixed points: pink cubes are the sets Ω; red dots are stable fixed points while the black dot is the unstable one. The time-scale parameters are $\tau_1 = \tau_2 = 0.01$, $\tau_3 = 1$, and in A, the other parameters are $a_1 = 2$, $a_2 = 1$, $b_1 = 1.2$, $b_2 = 1.7$, $c_1 = 2$, $c_2 = -3$, $d_1 = -4$, $d_2 = 5$, $e = 2$, $h = 6.6$, while in B $a_1 = 0.7$, $a_2 = 0.6$, $b_1 = 1.6$, $b_2 = 1.7$, $c_1 = 12$, $c_2 = -10$, $d_1 = -4$, $d_2 = 5$, $e = 0.6$, $h = 6$.

E Singular perturbation analysis

By a change of time $t' = \tau t$, we can rewrite the network motif dynamics without external inputs into

$$\begin{aligned} x_1' &= -a_1 x_1 + w_2 \phi(x_2) \\ x_2' &= -a_2 x_2 + w_1 \phi(x_1) \\ w_1' &= -b_1 w_1 + c_1 \phi(x_1)\phi(x_2) + d_1 \psi(z) \\ w_2' &= -b_2 w_2 + c_2 \phi(x_1)\phi(x_2) + d_2 \psi(z) \\ z' &= \tau(-\epsilon z + h\phi(x_1)\phi(x_2)), \end{aligned} \tag{12}$$

where $' = d/dt'$ denotes the differentiation with respect to t'. Because of the nature of the small value of τ, (1) (or (12)) indeed defines a perturbation problem. The *singular perturbation theory* [8] has been developed to solve such problems in the past few decades. In the following, we turn to the analysis of system (1) from the singular perturbation perspective.

By setting $\tau = 0$ in (12), we obtain the singular limit of the system, i.e.,

$$\begin{aligned} x_1' &= -a_1 x_1 + w_2 \phi(x_2) \\ x_2' &= -a_2 x_2 + w_1 \phi(x_1) \\ w_1' &= -b_1 w_1 + c_1 \phi(x_1)\phi(x_2) + d_1 \psi(z) \\ w_2' &= -b_2 w_2 + c_2 \phi(x_1)\phi(x_2) + d_2 \psi(z) \\ z' &= 0. \end{aligned} \tag{13}$$

In the above system, the derivative of z is zero. Intuitively, one can consider that the z-variable is fixed as in initial conditions, i.e., $z(t) = z_0 \in \mathbb{R}$. It results in the flowing truncated system

$$\begin{aligned} x_1' &= -a_1 x_1 + w_2 \phi(x_2) \\ x_2' &= -a_2 x_2 + w_1 \phi(x_1) \\ w_1' &= -b_1 w_1 + c_1 \phi(x_1)\phi(x_2) + d_1 \psi(z_0) \\ w_2' &= -b_2 w_2 + c_2 \phi(x_1)\phi(x_2) + d_2 \psi(z_0) \end{aligned} \tag{14}$$

The above system captures the dynamics on the neuronal layer, i.e., coupled rate-based RNN and Hebbian learning of synapses. Since z_0 is a constant in system (14), we take it as a non-dynamic parameter that can take different values.

As shown in Theorem 1, system (1) is bounded. Let $z_{\min}$ and $z_{\min}$ represent the minimum and maximum values that variable z can take in Ω. Under the assumption that $z(t)$ can span the whole admitted space in Ω, we have that $z_0 \in [z_{\min}, z_{\max}]$. As a consequence, if the dynamics of the neuronal layer exhibit critical changes, such as changes of the number and/or stability of the fixed points, we can say there exist bifurcations in (14) with respect to z_0, and these bifurcations are indeed induced by the self-slowly-varying astrocytic process.

In the following part, we will analyze the dynamics of the subsystems (14) in the spirit of the above idea.

Astrocytes regulate neural dynamics We visualize the change process of the fixed point set of system (14) as a consequence of the perturbation induced by the constant astrocytic signal. Since there are 4 variables in Eqs. (2), the first step will be reducing the dimension, otherwise, it is difficult to visualize these nullclines in

3D coordinates. By eliminating the variable w_2, we have

$$x_2 = \frac{w_1\phi(x_1)}{a_2} \tag{15a}$$

$$w_1 = \frac{c_1\phi(x_1)\phi(x_2) + d_1\psi(z_0)}{b_1} \tag{15b}$$

$$\frac{a_1x_1}{\phi(x_2)} = \frac{c_2\phi(x_1)\phi(x_2) + d_2\psi(z_0)}{b_2}. \tag{15c}$$

Note that the activation function $\psi_{\min} \leq \psi(z_0) \leq \psi_{\max}$. To examine the change of fixed points is equivalent to studying the change of the intersection of (15a)-(15c) as $\psi(z_0)$ varies in $[\psi_{\min}, \psi_{\max}]$. Recall that each equation of (15) defines a manifold in $(x_1, x_2, w_1) \in \tilde{\Omega}_{\text{motif}} \subset \mathbb{R}^3$. We can show these manifolds geometrically for given parameters, where Fig 4 displays the situations for $\psi(z_0) = \psi_{\min}$, $\psi(z_0) = 0$, and $\psi(z_0) = \psi_{\max}$ respectively under the parameter condition $a_1 = 0.3, a_2 = 0.4, b_1 = 1, b_2 = 0.5, c_1 = 6, c_2 = -5, d_1 = -2, d_2 = 3$.

In Fig4, when $\psi(z_0) \approx -1$ there is one fixed point (red dot); as $\psi(z_0)$ increases, the position of this fixed point changes accordingly. When $\psi(z_0) \approx 1$, another 2 fixed points exist. This means there is an increase of fixed point at a certain value of $\psi(z_0)$, and this is confirmed by the bifurcation diagram obtained with Matcont (see Fig 4D). It indicates that a branch of fixed points (red line) always exists. In contrast, the other branch of fixed points (blue line) exists when $\psi(z_0) \geq 0.7818$, but a saddle-node bifurcation occurs at $\psi(z_0) = 0.7818$ such that these two fixed points collide and annihilate each other. As the bifurcation happens in the limit $\tau \to 0$ of the original whole system, we call this process a *pseudo-bifurcation* resulting from the slow change of the astrocytic activity. And the neural dynamic behaviors are regulated by the astrocytic process in this top-down manner.

F Extended simulation results

In this section, we provide extra simulations that are complimentary to the results in the main text.

F.1 Regrets and converging time

Fig 5 shows the details of each method in the learning procedure. As shown in the plots, the regrets of every method are dense at the beginning because the

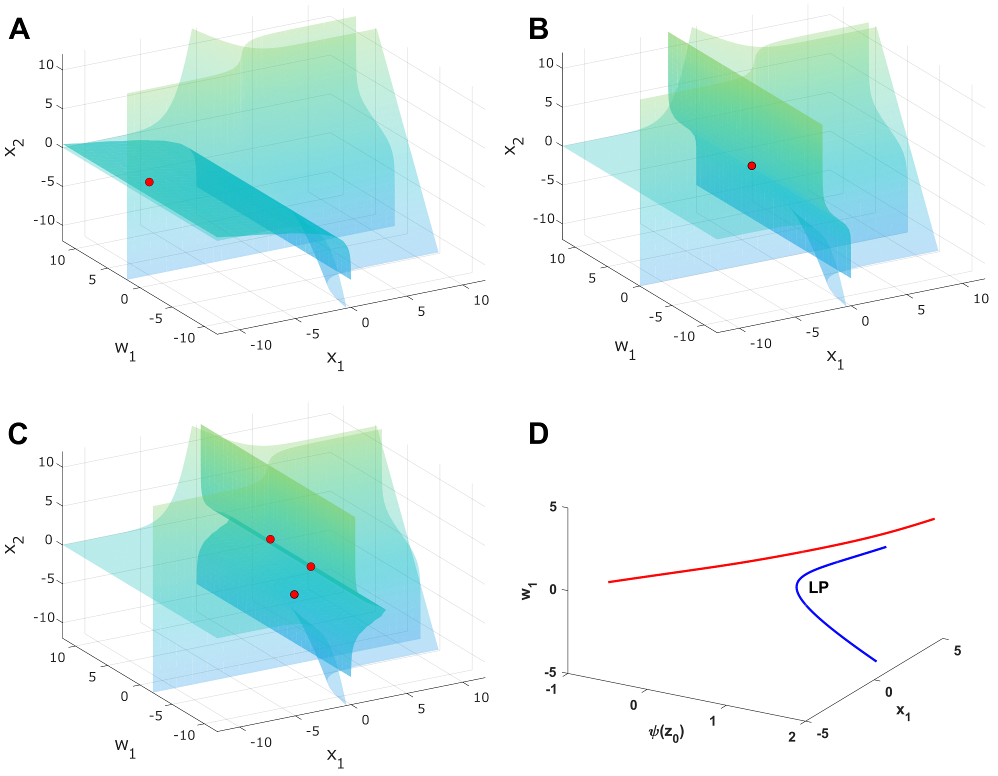

Figure 4: The fixed points (red dots) of the neural subsystem for different values of $\psi(z_0)$: A. $\psi(z_0) \approx -1$, B. $\psi(z_0) = 0$, C. $\psi(z_0) \approx 1$ In D, LP denotes the bifurcation point.

agent needs to explore the environment. After enough time, the agent can make the optimal actions such that there are no more regrets. It is observed that the neuron-astrocyte method does not generate regrets after about 2000 trials while other methods still give rise to regrets in the remaining trials. Therefore, the neuron-astrocyte method takes the shortest time to converge. In the right plot, we can see that the neuron-astrocyte method accounts for a medium amount of asymptotic cumulative regret among all the methods, while TS method has the lowest and UCB method has the highest asymptotic cumulative regret.

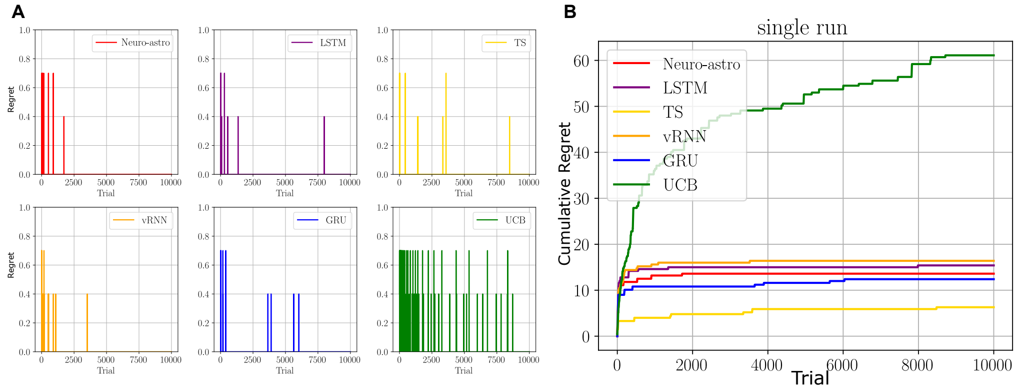

Figure 5: Regrets per trial and cumulative regrets of each method. A shows the regrets per trial for different methods, while B shows the cumulative regrets over trials.

F.2 Robustness analysis in diverse stationary bandits

We conduct a robustness analysis of all methods under various conditions of arm probabilities, specifically $(\mu_1, \mu_2, \mu_3) = (0.6 - \lambda, 0.6, 0.6 + \lambda)$ with $0 < \lambda < 0.4$. The UCB method is less competitive and excluded from this comparison due to its significantly larger regrets. As λ decreases, the bandit becomes more challenging due to the arms' probabilities converging. To vary the difficulty of the bandit tasks, we change λ from 0.38 to 0.02 (see Fig 6). It is recognized that the neuron-astrocyte method performs similarly to other RNN-based methods under larger λ values. However, our method tends to exhibit better and more robust performance in more challenging bandits in contrast to others, which experience a decline in performance.

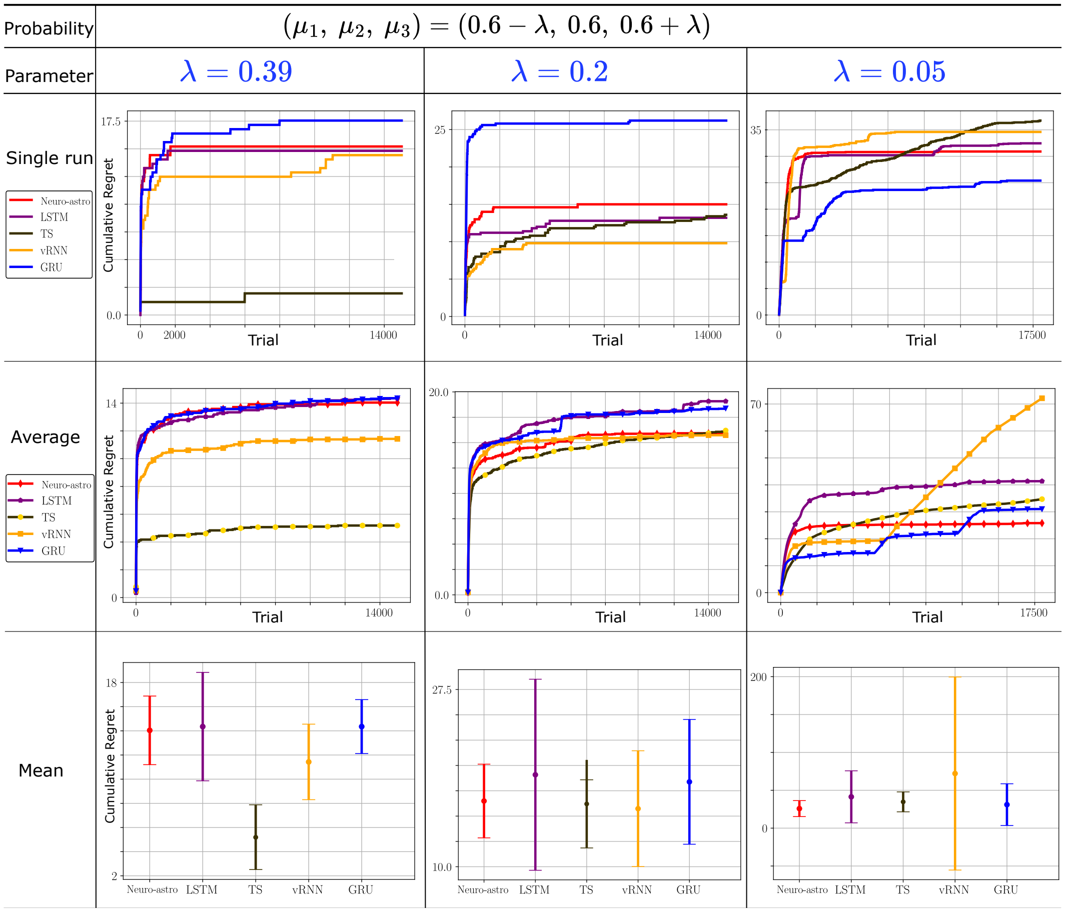

Figure 6: The learning performance robustness of each method is examined under different conditions of arm probabilities.

F.3 Detailed performance comparison in non-stationary bandits

Fig 7 gives a comprehensive comparison of different methods in the non-stationary bandits. The neuron-astrocyte method consistently attains the lowest and maintains near-stationary asymptotic cumulative regret, both in single and multiple runs. In contrast, other methods struggle to adapt to evolving environments, resulting in steadily increasing regrets. This result is corroborated in experiments of both the flip-flop and smooth-changing non-stationary bandits.

F.4 Time-scale separation impact

The time-scale parameter has a mild impact on the learning performance of the stationary bandit. When $\tau = 0.1$, the average cumulative regret is the smallest. It is also noted that the algorithm becomes more stable as the standard deviation of the final regrets becomes smaller as τ decreases. The time-scale separation has important influence on the learning performance for the non-stationary bandit. If there is no difference between the time-scales, i.e., $\tau = 1$, the cumulative regrets will keep increasing and cannot reach a final stationary value for all runs, which means the agent is not able to adapt to the changing environments. When $\tau = 0.1$, the agent can achieve stationary cumulative regrets occasionally over multiple runs; if $\tau \leq 0.01$, the agent can always achieve stationary asymptotic cumulative regret.

F.5 Flexible generalization to bandit tasks with different number of actions

In previous demonstrations, we have simulated the bandit tasks with only 3 actions. Here, we show that the neuron-astrocyte networks and the designed learning algorithm can be easily generalized to other cases by providing an illustrative example involving an 8-action Bernoulli bandit. In the stationary situation, the means for the actions are fixed as $\mu = (0.1, 0.2, 0.3, 0.4, 0.6, 0.7, 0.8, 0.9)$. The non-stationary version is designed with the means changing abruptly from μ to $1 - \mu$ every 5000 trials.

To accommodate the 8 actions of the bandit, the neuron-astrocyte learning algorithm can be modified by simply expanding the dimension of the neuron-astrocyte module's outputs to 8, while leaving other settings unchanged. It is validated from simulations that our method can solve these even challenging tasks

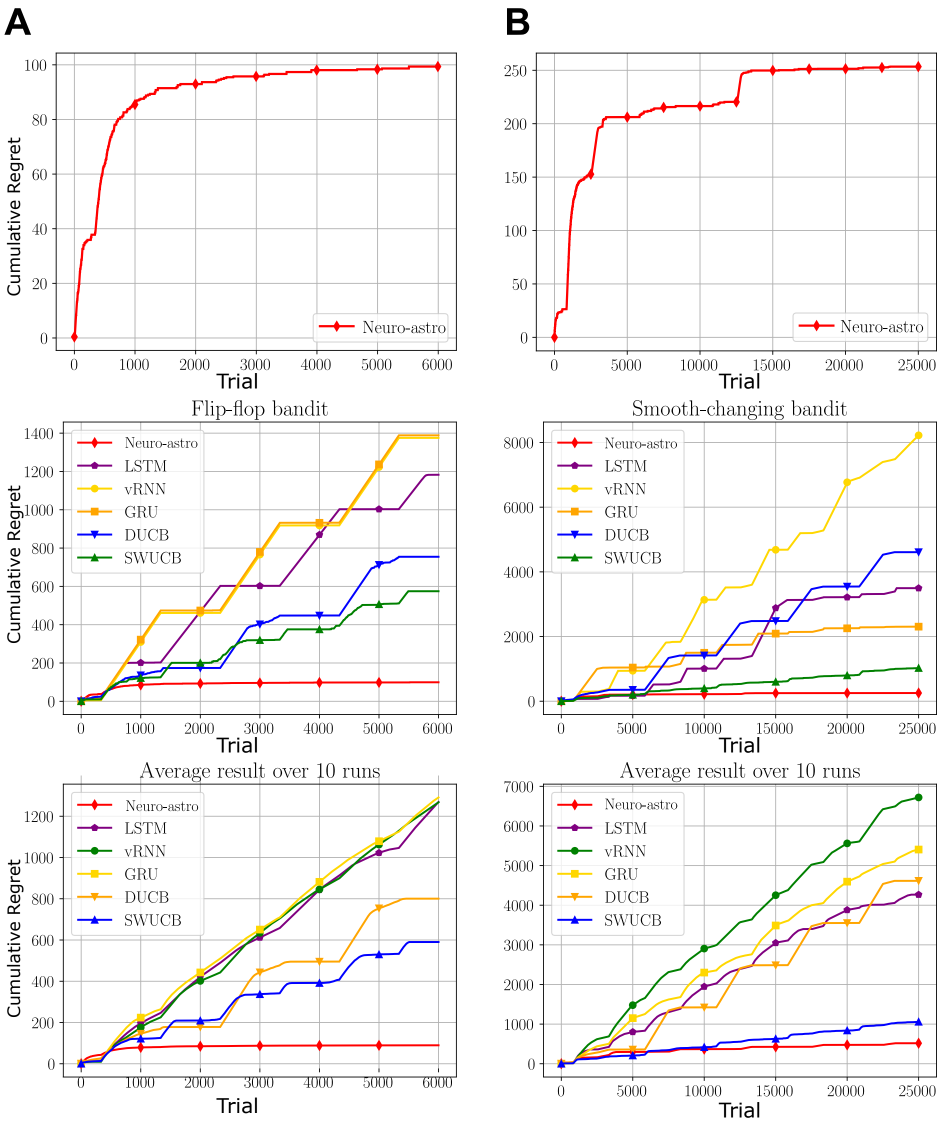

Figure 7: Learning performance in non-stationary Bernoulli bandits. For flip-flop (panel A) and smooth-changing (panel B) cases, the cumulative regrets of different methods (neuron-astrocyte, LSTM, vRNN, GRU, DUCB, SWUCB) are shown for the single simulation and the average of 10 runs. The hyperparameters of DUCB and SWUCB have been carefully tuned to optimize their performance.

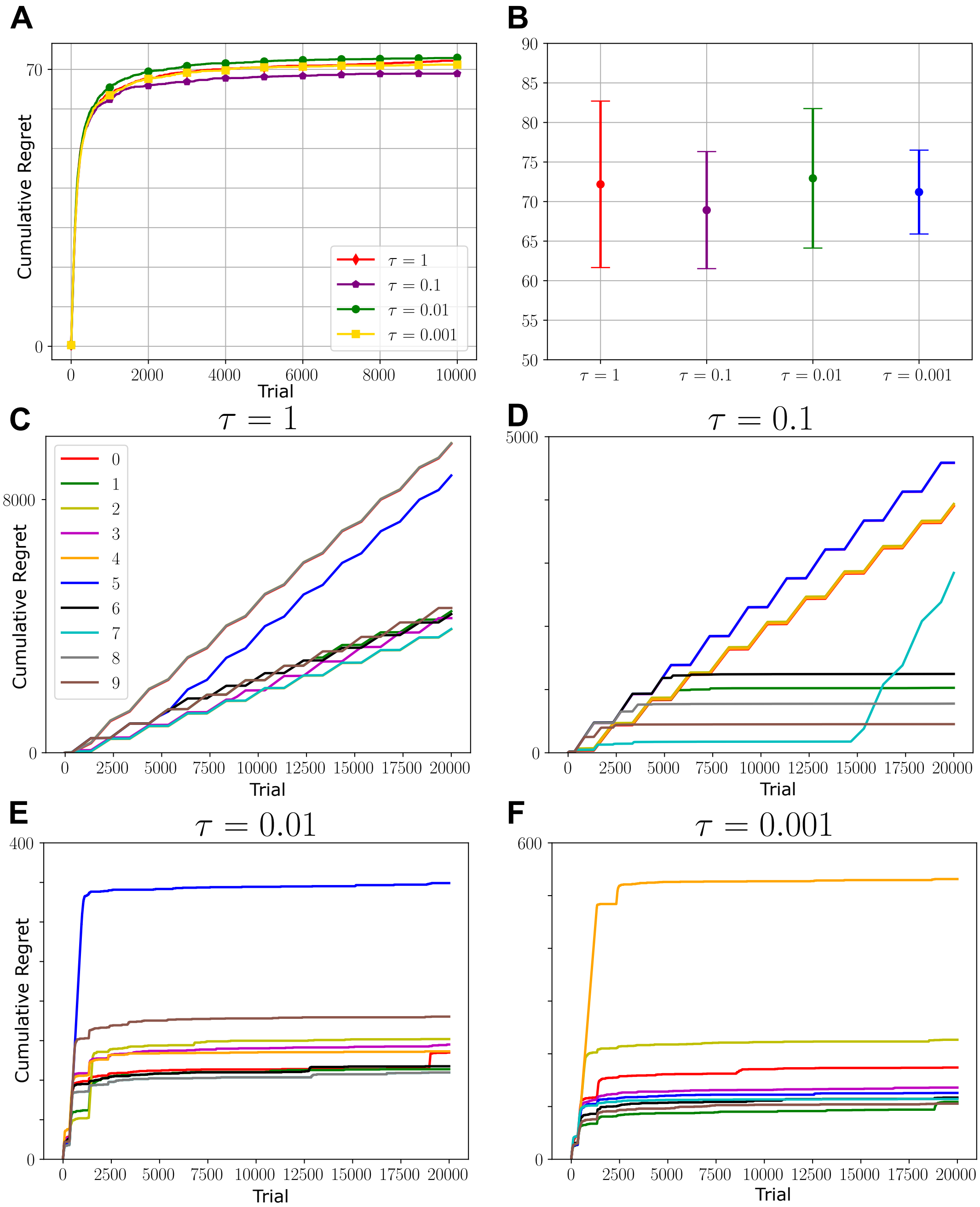

Figure 8: A is the average cumulative regrets over 10 runs of the neuron-astrocyte method with different τ in the stationary Bernoulli bandit, while B displays the mean and standard deviation of the asymptotic cumulative regrets. C-F show the cumulative regrets in each individual run of the neuron-astrocyte method with different τ in the flip-flop switching bandit.

very well in both stationary and non-stationary situations, and its learning performance is superior in comparison with other methods (as shown in Fig 9).

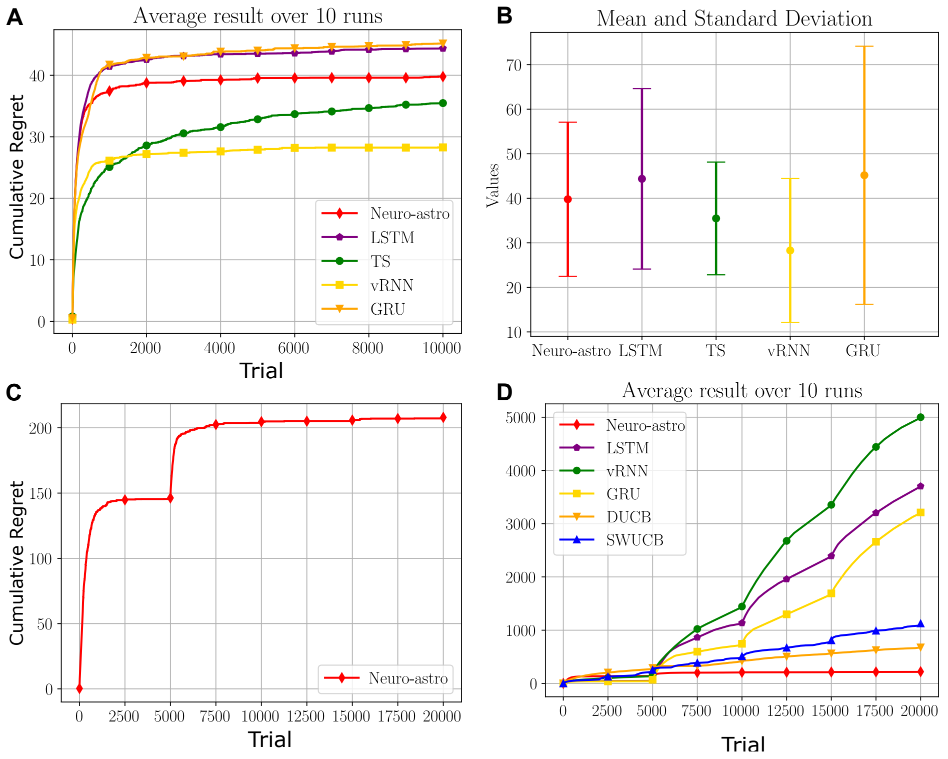

Figure 9: A. The plots show the average results and the means and standard deviations of the asymptotic cumulative regret of different methods in the stationary bandit with 8 actions. B. The left plot is the result of a single simulation in this non-stationary bandit, while the right shows the average cumulative results of the neuron-astrocyte method in comparison with other methods (again, DUCB and SWUCB methods have been tuned carefully).

F.6 Impact of astrocyte-neuron ratio on learning performance

We provide now a more complete characterization of the effect of different ratios between neurons and astrocytes. Let $R = \frac{\#astrocytes}{\#neurons}$. As shown in Fig. 10, the ratios have important influence on the learning performance in both stationary and non-stationary bandits scenarios. In the stationary case, an increased ratio

positively influences learning performance, leading to a reduction in asymptotic cumulative regret as more astrocytes are introduced. However, the dynamic nature of the flip-flop bandit introduces a subtle impact of the ratio: optimal cumulative regret occurs at *intermediate* ratios, whereas both excessively low and high ratios detrimentally affect learning by increasing regret. This outcome suggests an interesting interpretation: in non-stationary environments, the network requires appropriate exploration of the environment. Too few astrocytes hinder guidance for exploration, while an excess of astrocytes imposes overly strong regulation, limiting neural network flexibility. It turns out that the optimal ratio is around 7/10. In biology, the ratio of astrocytes to neurons is believed to be between 1:1.5 and 1:2, hence our modeling observation provides an interesting interpretation of the biology in this context.

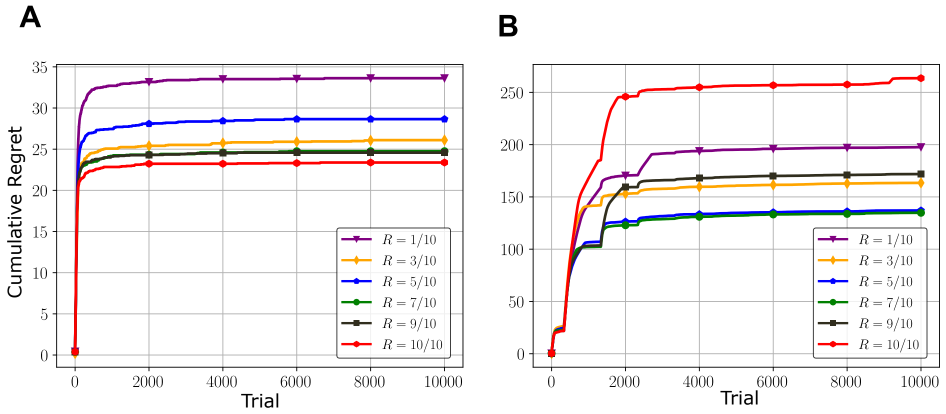

Figure 10: Learning performance of neuro-astrocyte networks with different astrocytes to neurons ratios ranging from $1/10$ to $10/10$. A is for the stationary bandit, while B is for the flip-flop non-stationary bandit.

F.7 Alternative model via potassium dynamics

Regarding slow dynamics potentially arising from passive potassium efflux. we have considered a null model of sorts, which was called the 'potassium only'

model, with slow passive potassium dynamics [9] described as:

$$\dot{x}_i = -a_i x_i + \sum_j^n w_{ij}\phi(x_j) - \lambda_i y_i + u_i$$

$$\tau_p \dot{y}_i = \sum_j^n p_j \phi(x_j),$$

where y_i denotes the potassium activity. This model enacts a slow time-scale, but without the nested feedback loop structure that we believe is key to neural-astrocyte interaction.

We implement the potassium-only model into the learning algorithm. After the same training procedure as in our primary results, we are able to show that this null model can only learn the stationary (but not the non-stationary) version of the task (see Fig. 11). This lends credence to the idea that the unique inter-activity properties of astrocytes with neurons are important to the purposes and hypothesized functional benefits.

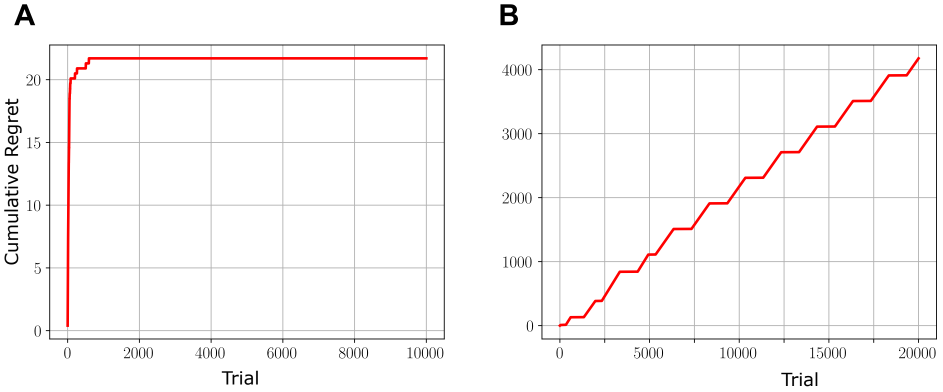

Figure 11: Cumulative regrets of the agent employing a neural network with passive potassium dynamics, where A is for the stationary bandit and B is for the flip-flop non-stationary bandit.

F.8 Heterogneous astrocytic modulation

We examine different projections of astrocytic and synaptic activities of the same trained model as in the main result. A second principal component (different from the one in Fig 4F in the main text) of astrocytic activities shows the sign

of astrocytic modulation changes across trials and contexts (see Fig 12), which directly suggests the astrocytic modulation on meta-plasticity is heterogeneous.

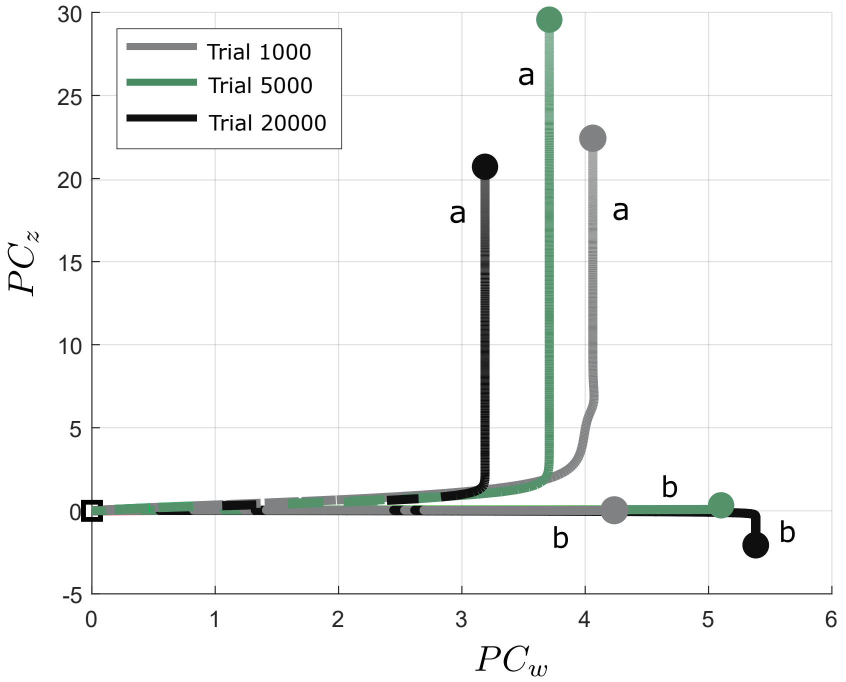

Figure 12: Additional projections of astrocyte and synaptic activity for both contexts (indicated as a and b) in the early, middle, and late phases of learning, highlighting the formation of distinct synaptic weight trajectories

References

[1] Boccaletti S, Bianconi G, Criado R, del Genio CI, Gómez-Gardeñes J, Romance M, et al. The structure and dynamics of multilayer networks. Physics Reports. 2014;544:1–122.

[2] Perko L. Differential equations and dynamical systems. New York: Springer Science & Business Media; 2013.

[3] Khalil HK. Nonlinear Systems. Hoboken: Prentice Hall; 1996.

[4] Centorrino V, Bullo F, Russo G. Contraction Analysis of Hopfield Neural Networks with Hebbian Learning. In: IEEE 61st Conference on Decision and Control; 2022. p. 622–627.

[5] Wiggins S, Holmes P, John F, Jager W, Marsden JE, Sirovich L, et al. Introduction to Applied Nonlinear Dynamical Systems and Chaos. New York: Springer; 1990.

[6] Karamardian S. Fixed points: algorithms and applications. Cambridge: Academic press; 2014.

[7] Chicone C. Ordinary Differential Equations with Applications. Texts in Applied Mathematics. New York: Springer New York; 2008.

[8] Kuehn C. Multiple Time Scale Dynamics. Applied Mathematical Sciences. New York: Springer; 2015.

[9] Odette LL, Newman EA. Model of potassium dynamics in the central nervous system. Glia. 1988;1(3):198–210.
